# Selective Preference of Parallel DNA Triplexes Is Due to the Disruption of Hoogsteen Hydrogen Bonds Caused by the Severe Nonisostericity between the G*GC and T*AT Triplets

**DOI:** 10.1371/journal.pone.0152102

**Published:** 2016-03-24

**Authors:** Gunaseelan Goldsmith, Thenmalarchelvi Rathinavelan, Narayanarao Yathindra

**Affiliations:** 1 Institute of Bioinformatics and Applied Biotechnology, Biotech Park, Electronics City Phase I, Bangalore, India; 2 Manipal University, Manipal, India; Wake Forest University, UNITED STATES

## Abstract

Implications of DNA, RNA and RNA.DNA hybrid triplexes in diverse biological functions, diseases and therapeutic applications call for a thorough understanding of their structure-function relationships. Despite exhaustive studies mechanistic rationale for the discriminatory preference of parallel DNA triplexes with G_*_GC & T_*_AT triplets still remains elusive. Here, we show that the highest nonisostericity between the G_*_GC & T_*_AT triplets imposes extensive stereochemical rearrangements contributing to context dependent triplex destabilisation through selective disruption of Hoogsteen scheme of hydrogen bonds. MD simulations of nineteen DNA triplexes with an assortment of sequence milieu reveal for the first time fresh insights into the nature and extent of destabilization from a single (non-overlapping), double (overlapping) and multiple pairs of nonisosteric base triplets (NIBTs). It is found that a solitary pair of NIBTs, feasible either at a G_*_GC/T_*_AT or T_*_AT/G_*_GC triplex junction, does not impinge significantly on triplex stability. But two overlapping pairs of NIBTs resulting from either a T_*_AT or a G_*_GC interruption disrupt Hoogsteen pair to a noncanonical mismatch destabilizing the triplex by ~10 to 14 kcal/mol, implying that their frequent incidence in multiples, especially, in short sequences could even hinder triplex formation. The results provide (i) an unambiguous and generalised mechanistic rationale for the discriminatory trait of parallel triplexes, including those studied experimentally (ii) clarity for the prevalence of antiparallel triplexes and (iii) comprehensive perspectives on the sequence dependent influence of nonisosteric base triplets useful in the rational design of TFO’s against potential triplex target sites.

## Introduction

It is well documented that Watson and Crick paired DNA duplex interacts with sequence specific oligonucleotides to form triple helices stabilised by either Hoogsteen (parallel triplex) or reverse Hoogsteen (antiparallel triplex) pair of hydrogen bonds. Evidences for involvement of triple helices in biological processes come from their participation in regulation of gene expression [1, 2], DNA damage and repair [3, 4], RNA processing and folding [5, 6, 7] and chromatin organization [8]. Triplexes are also known to impair DNA polymerization and influence DNA recombination process [9]. Base triple interactions crucial for function are shown to be present in pseudoknots in telomerase RNA [10] and in transcripts during programmed ribosomal frame shifting in viruses like SARS coronavirus [11]. Most recently, intramolecularly folded RNA triplexes are shown to be formed in the highly abundant and up-regulated long noncoding transcripts such as metastasis associated lung adenocarcinoma transcript 1 (MALAT1) and others to evade exonuclease action [12, 13]. Formation of RNA.DNA hybrid triplexes involving noncoding RNA found to repress genes like DHFR, TGF-β and MAT2A has further enhanced the biological importance of nucleic acid triplexes [14, 15, 16]. Existence of triplexes *in vivo* is supported by the discovery of triplex unwinding helicases [17, 18], triplex-specific antibodies [19, 20] and other endogenous triplex specific proteins [21–24]. Association of triple helices with colorectal cancer [25], neurodegenerative disorder Friedreich’s ataxia [26] and a number of inherited as well as acquired human diseases [27] further underscore their profound significance.

DNA triplexes have evoked potential technological applications which include creating high quality DNA vectors for human gene therapy [28], nanomachines for monitoring intracellular pH gradient [29], molecular switches [30,31,32], and for developing drug delivery systems [33,34]. They have also been used as biosensors for the recognition and analysis of toxic metal ions [35], SNPs [36], DNA methylation [37] and cancer cells [38]. Quite recently, DNA triplex formation process has been exploited in developing PCR based biosensor for detection of pathogens [39], high-throughput assays for measuring activity of DNA topoisomerases and other enzymes involved in DNA topology modification [40], controlled assembly of liposomes [41] and as modular probes for DNA detection [42]. Recognition of abundant putative triplex target sites in both prokaryotic and eukaryotic genomes [43, 44, 45], together with new experimental approaches for identification of triple helices [46–50] and development of algorithm/databases for analysis of triplex target sites [51, 52, 53] highlight their increasing relevance in the post genomic era. Several reviews detailing the biological significance [2, 54–60] and potential therapeutic applications [61] of triplexes have been appearing to underscore their importance. Notwithstanding, structural information on triple helices, specifically those addressing the issue of base triplet nonisostericity and their effects, crucial in the design of Triplex forming Oligonucleotides (TFOs) for targeting duplex is rather inadequate.

It is well recognised that TFOs rich in (i) T & C^+^, (ii) G & A, (iii) G & T can interact with purine rich strand of DNA forming a pair of hydrogen bonds to result in T_*_AT, C^+^_*_GC, G_*_GC and A_*_AT base triplets. Predictably, T_*_AT & C^+^_*_GC triplets are isosteric (structurally alike) like the Watson and Crick pairs, while the other triplet pair combinations such as T_*_AT & G_*_GC, T_*_AT & G_*_GC and A_*_AT & G_*_GC are nonisosteric (structurally dissimilar) [56, 62]. While T & C rich TFOs favour parallel triplex [63, 64], those with G & A favour antiparallel triplex [65, 66]. Although TFOs with G & T form both parallel and antiparallel triplexes, they predominantly favour the latter [67–70]. Interestingly, both intramolecular [67, 71] and intermolecular [62, 72–74] parallel triplexes containing a limited number of G_*_GC & T_*_AT triplet pairs are observed. They are shown to be effective in interfering with biological functions in several instances. For instance, GT rich 38-mer and 15-mer TFOs inhibit transcription of HIV-1 in infected human cells [75] and replication in SV40 containing plasmid in COS-1 cells [76] respectively, through formation of parallel triplexes. On the other hand, the 22-mer TFO with 10 T_*_AT & G_*_GC juxtapositions, and the 36-mer TFO with 21 T_*_AT & G_*_GC juxtapositions, targeted against the promoter region of human Ki-*ras* [77] and the human epidermal growth factor receptor gene [78] respectively, are unable to form parallel triplex, but forms only antiparallel triplex. Seemingly then, selective ability to form parallel triplex appears to be determined by the number of G_*_GC & T_*_AT juxtapositions, their larger incidences, thwarting parallel triplex formation. While this may be notionally attributed to nonisosteric traits between them, their quantitative estimates and precise mechanistic effects on the ability and stability for triplex formation are obscure.

**Fig 1:**
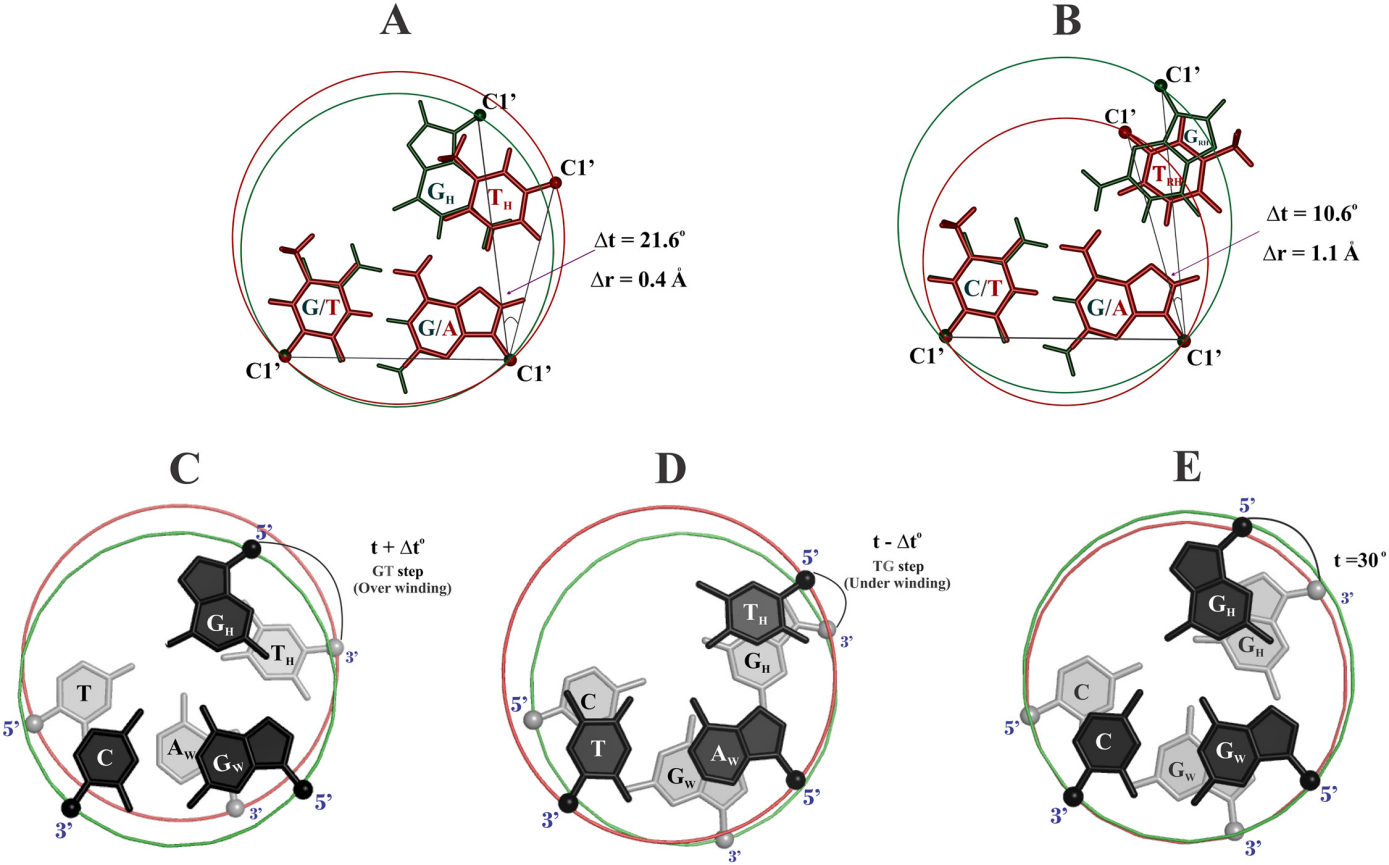
Nature, degree of base triplet nonisostericity and their consequence.

A cursory glance at the superposition of G_*_GC and T_*_AT triplet pairs (Fig 1A and 1B), readily point to the existence of an intrinsically large nonisostericity (defined by the residual twist angle, Δt°) in parallel compared to antiparallel orientation. We argue that this might be responsible in imparting differential mechanistic influence in parallel vis-à-vis antiparallel triplexes causing selective preference of the former. To probe this, extensive MD simulations of a variety of parallel and anti-parallel DNA triplexes formed by these triplets, in different sequence contexts (Sequences 1–19 in Table 1) have been carried out. Results have yielded clear insights into the role of nonisostericity on triplex stability, stereochemical reasoning for the prevalence of antiparallel triplex and selective preference for parallel triplex. These should aid in comprehensive understanding of sequence dependent structure of nucleic acid triplexes and their implication in biological processes.

**Table 1:** DNA triplexes considered for the study.

Sequence No. (Oligomer size)	DNA Triplex Sequence	No. of GGC/TAT triplets	No. of GT/TG Steps	No. and type of NIBT pairs	Simulation Time (ns)	Binding Free energy (kcal/mol)
**1.** (15-mer)	**5’ C**_**1**_***T***_***2***_**C**_**3**_***T***_***4***_**C**_**5**_***T***_***6***_**C**_**7**_***T***_***8***_**C**_**9**_***T***_***10***_**C**_**11**_***T***_***12***_**C**_**13**_***T***_***14***_**C**_**15**_	8/7	7GT 7TG	14 Overlapping	250	-9.4
	**3’ G**_**30**_***A***_***29***_**G**_**28**_***A***_***27***_**G**_**26**_***A***_***25***_**G**_**24**_***A***_***23***_**G**_**22**_***A***_***21***_**G**_**20**_***A***_***19***_**G**_**18**_***A***_***17***_**G**_**16**_					
	**3’ G**_**45**_***T***_***44***_**G**_**43**_***T***_***42***_**G**_**41**_***T***_***40***_**G**_**39**_***T***_***38***_**G**_**37**_***T***_***36***_**G**_**35**_***T***_***34***_**G**_**33**_***T***_***32***_**G**_**31**_**TFO**					
**2.** (15-mer) ***Antiparallel***	**5’ C**_**1**_***T***_***2***_**C**_**3**_***T***_***4***_**C**_**5**_***T***_***6***_**C**_**7**_***T***_***8***_**C**_**9**_***T***_***10***_**C**_**11**_***T***_***12***_**C**_**13**_***T***_***14***_**C**_**15**_	8/7	7GT 7TG	14 Overlapping	100	-70.8
	**3’ G**_**30**_***A***_***29***_**G**_**28**_***A***_***27***_**G**_**26**_***A***_***25***_**G**_**24**_***A***_***23***_**G**_**22**_***A***_***21***_**G**_**20**_***A***_***19***_**G**_**18**_***A***_***17***_**G**_**16**_					
	**5’ G**_**31**_***T***_***32***_**G**_**33**_***T***_***34***_**G**_**35**_***T***_***36***_**G**_**37**_***T***_***38***_**G**_**39**_***T***_***40***_**G**_**41**_***T***_***42***_**G**_**43**_***T***_***44***_**G**_**45**_**TFO**					
**3.** (11-mer)	**5’ *T***_***1***_***T***_***2***_***T***_***3***_***T***_***4***_***T***_***5***_***T***_***6***_***T***_***7***_***T***_***8***_***T***_***9***_***T***_***10***_***T***_***11***_	11	0	homopolymer	100	-63.6
	**3’ *A***_***22***_***A***_***21***_***A***_***20***_***A***_***19***_***A***_***18***_***A***_***17***_***A***_***16***_***A***_***15***_***A***_***14***_***A***_***13***_***A***_***12***_					
	**3’ *T***_***33***_***T***_***32***_***T***_***31***_***T***_***30***_***T***_***29***_***T***_***28***_***T***_***27***_***T***_***26***_***T***_***25***_***T***_***24***_***T***_***23***_**TFO**					
**4.** (11-mer)	**5’ *T***_***1***_***T***_***2***_***T***_***3***_***T***_***4***_***T***_***5***_**C**_**6**_***T***_***7***_***T***_***8***_***T***_***9***_***T***_***10***_***T***_***11***_	1/10	1TG 1GT	2 Overlapping	100	-53.5
	**3’ *A***_***22***_***A***_***21***_***A***_***20***_***A***_***19***_***A***_***18***_**G**_**17**_***A***_***16***_***A***_***15***_***A***_***14***_***A***_***13***_***A***_***12***_					
	**3’ *T***_***33***_***T***_***32***_***T***_***31***_***T***_***30***_***T***_***29***_**G**_**28**_***T***_***27***_***T***_***26***_***T***_***25***_***T***_***24***_***T***_***23***_**TFO**					
**5.** (11-mer)	**5’ *T***_***1***_***T***_***2***_***T***_***3***_**C**_**4**_***T***_***5***_***T***_***6***_***T***_***7***_**C**_**8**_***T***_***9***_***T***_***10***_***T***_***11***_	2/9	2TG 2GT	4 Overlapping	100	-40.2
	**3’ *A***_***22***_***A***_***21***_***A***_***20***_**G**_**19**_***A***_***18***_***A***_***17***_***A***_***16***_**G**_**15**_***A***_***14***_***A***_***13***_***A***_***12***_					
	**3’ *T***_***33***_***T***_***32***_***T***_***31***_**G**_**30**_***T***_***29***_***T***_***28***_***T***_***27***_**G**_**26**_***T***_***25***_***T***_***24***_***T***_***23***_**TFO**					
**6.** (11-mer)	**5’ *T***_***1***_***T***_***2***_**C**_**3**_***T***_***4***_***T***_***5***_***C***_***6***_***T***_***7***_***T***_***8***_**C**_**9**_***T***_***10***_***T***_***11***_	3/9	3TG 3GT	6 Overlapping	100	-25.9
	**3’ *A***_***22***_***A***_***21***_**G**_**20**_***A***_***19***_***A***_***18***_***G***_***17***_***A***_***16***_***A***_***15***_**G**_**14**_***A***_***13***_***A***_***12***_					
	**3’ *T***_***33***_***T***_***32***_**G**_**31**_***T***_***30***_***T***_***29***_***G***_***28***_***T***_***27***_***T***_***26***_**G**_**25**_***T***_***24***_***T***_***23***_**TFO**					
**7.** (11-mer)	**5’ C**_**1**_**C**_**2**_**C**_**3**_**C**_**4**_**C**_**5**_**C**_**6**_**C**_**7**_**C**_**8**_**C**_**9**_**C**_**10**_**C**_**11**_	11	0	homopolymer	100	-62.0
	**3’ G**_**22**_**G**_**21**_**G**_**20**_**G**_**19**_**G**_**18**_**G**_**17**_**G**_**16**_**G**_**15**_**G**_**14**_**G**_**13**_**G**_**12**_					
	**3’ G**_**33**_**G**_**32**_**G**_**31**_**G**_**30**_**G**_**29**_**G**_**28**_**G**_**27**_**G**_**26**_**G**_**25**_**G**_**24**_**G**_**23**_**TFO**					
**8.** (11-mer)	**5’ C**_**1**_**C**_**2**_**C**_**3**_**C**_**4**_**C**_**5**_***T***_***6***_**C**_**7**_**C**_**8**_**C**_**9**_**C**_**10**_**C**_**11**_	10/1	1GT 1TG	2 Overlapping	100	-50.6
	**3’ G**_**22**_**G**_**21**_**G**_**20**_**G**_**19**_**G**_**18**_***A***_***17***_**G**_**16**_**G**_**15**_**G**_**14**_**G**_**13**_**G**_**12**_					
	**3’ G**_**33**_**G**_**32**_**G**_**31**_**G**_**30**_**G**_**29**_***T***_***28***_**G**_**27**_**G**_**26**_**G**_**25**_**G**_**24**_**G**_**23**_**TFO**					
**9.** (11-mer)	**5’ C**_**1**_**C**_**2**_**C**_**3**_***T***_***4***_**C**_**5**_**C**_**6**_**C**_**7**_***T***_***8***_**C**_**9**_**C**_**10**_**C**_**11**_	10/1	2GT 2TG	4 Overlapping	100	-39.3
	**3’ G**_**22**_**G**_**21**_**G**_**20**_***A***_***19***_**G**_**18**_**G**_**17**_**G**_**16**_***A***_***15***_**G**_**14**_**G**_**13**_**G**_**12**_					
	**3’ G**_**33**_**G**_**32**_**G**_**31**_***T***_***30***_**G**_**29**_**G**_**28**_**G**_**27**_***T***_***26***_**G**_**25**_**G**_**24**_**G**_**23**_**TFO**					
**10.** (15-mer)	**5’ *T***_***1***_***T***_***2***_***T***_***3***_***T***_***4***_***T***_***5***_***T***_***6***_***T***_***7***_**C**_**8**_**C**_**9**_**C**_**10**_**C**_**11**_**C**_**12**_**C**_**13**_**C**_**14**_**C**_**15**_	8/7	1GT	1 Non-Overlapping	100	-71.6
	**3’ *A***_***30***_***A***_***29***_***A***_***28***_***A***_***27***_***A***_***26***_***A***_***25***_***A***_***24***_**G**_**23**_**G**_**22**_**G**_**21**_**G**_**20**_**G**_**19**_**G**_**18**_**G**_**17**_**G**_**16**_					
	**3’ *T***_***45***_***T***_***44***_***T***_***43***_***T***_***42***_***T***_***41***_***T***_***40***_***T***_***39***_**G**_**38**_**G**_**37**_**G**_**36**_**G**_**35**_**G**_**34**_**G**_**33**_**G**_**32**_**G**_**31**_**TFO**					
**11.** (15-mer)	**5’ C**_**1**_**C**_**2**_**C**_**3**_**C**_**4**_**C**_**5**_**C**_**6**_**C**_**7**_**C**_**8**_***T***_***9***_***T***_***10***_***T***_***11***_***T***_***12***_***T***_***13***_***T***_***14***_***T***_***15***_	8/7	1TG	1 Non-Overlapping	100	-78.4
	**3’ G**_**30**_**G**_**29**_**G**_**28**_**G**_**27**_**G**_**26**_**G**_**25**_**G**_**24**_**G**_**23**_***A***_***22***_***A***_***21***_***A***_***20***_***A***_***19***_***A***_***18***_***A***_***17***_***A***_***16***_					
	**3’ G**_**45**_**G**_**44**_**G**_**43**_**G**_**42**_**G**_**41**_**G**_**40**_**G**_**39**_**G**_**38**_***T***_***37***_***T***_***36***_***T***_***35***_***T***_***34***_***T***_***33***_***T***_***32***_***T***_***31***_**TFO**					
**12.** (15-mer)	**5’ *T***_***1***_***T***_***2***_***T***_***3***_**C**_**4**_**C**_**5**_**C**_**6**_**C**_**7**_**C**_**8**_**C**_**9**_**C**_**10**_**C**_**11**_***T***_***12***_***T***_***13***_***T***_***14***_***T***_***15***_	8/7	1TG 1GT	2 Non-Overlapping	100	-57.2
	**3’ *A***_***30***_***A***_***29***_***A***_***28***_**G**_**27**_**G**_**26**_**G**_**25**_**G**_**24**_**G**_**23**_**G**_**22**_**G**_**21**_**G**_**20**_***A***_***19***_***A***_***18***_***A***_***17***_***A***_***16***_					
	**3’ *T***_***45***_***T***_***44***_***T***_***43***_**G**_**42**_**G**_**41**_**G**_**40**_**G**_**39**_**G**_**38**_**G**_**37**_**G**_**36**_**G**_**35**_***T***_***34***_***T***_***33***_***T***_***32***_***T***_***31***_**TFO**					
**13.** (15-mer)	**5’ C**_**1**_**C**_**2**_**C**_**3**_**C**_**4**_***T***_***5***_***T***_***6***_***T***_***7***_***T***_***8***_***T***_***9***_***T***_***10***_***T***_***11***_**C**_**12**_**C**_**13**_**C**_**14**_**C**_**15**_	8/7	1GT 1TG	2 Non-Overlapping	100	-55.8
	**3’ G**_**30**_**G**_**29**_**G**_**28**_**G**_**27**_***A***_***26***_***A***_***25***_***A***_***24***_***A***_***23***_***A***_***22***_***A***_***21***_***A***_***20***_**G**_**19**_**G**_**18**_**G**_**17**_**G**_**16**_					
	**3 ‘ G**_**45**_**G**_**44**_**G**_**43**_**G**_**42**_***T***_***41***_***T***_***40***_***T***_***39***_***T***_***38***_***T***_***37***_***T***_***36***_***T***_***35***_**G**_**34**_**G**_**33**_**G**_**32**_**G**_**31**_**TFO**					
**14.** (15-mer)	**5’ *T***_***1***_***T***_***2***_**C**_**3**_**C**_**4**_**C**_**5**_**C**_**6**_***T***_***7***_***T***_***8***_***T***_***9***_**C**_**10**_**C**_**11**_**C**_**12**_**C**_**13**_***T***_***14***_***T***_***15***_	8/7	2TG 2GT	4 Non-Overlapping	100	-44.3
	**3’ *A***_***30***_***A***_***29***_**G**_**28**_**G**_**27**_**G**_**26**_**G**_**25**_***A***_***24***_***A***_***23***_***A***_***22***_**G**_**21**_**G**_**20**_**G**_**19**_**G**_**18**_***A***_***17***_***A***_***16***_					
	**3 ‘ *T***_***45***_***T***_***44***_**G**_**43**_**G**_**42**_**G**_**41**_**G**_**40**_***T***_***39***_***T***_***38***_***T***_***37***_**G**_**36**_**G**_**35**_**G**_**34**_**G**_**33**_***T***_***32***_***T***_***31***_**TFO**					
**15.** (25-mer)	**5’ C**_**1**_**C**_**2**_**C**_**3**_**C**_**4**_***T***_***5***_***T***_***6***_***T***_***7***_***T***_***8***_***T***_***9***_**C**_**10**_**T**_**11**_**T**_**12**_**T**_**13**_**T**_**14**_**T**_**15**_**C**_**16**_**C**_**17**_**C**_**18**_**C**_**19**_**C**_**20**_**T**_**21**_**C**_**22**_**C**_**23**_**C**_**24**_**C**_**25**_	14/11	3GT 3TG	4 Overlapping & 2 Non-Overlapping	100	-81.2
	**3’G**_**50**_**G**_**49**_**G**_**48**_**G**_**47**_***A***_***46***_***A***_***45***_***A***_***44***_***A***_***43***_***A***_***42***_***G***_***41***_***A***_***40***_***A***_***39***_***A***_***38***_***A***_***37***_***A***_***36***_**G**_**35**_**G**_**34**_**G**_**33**_**G**_**32**_**G**_**31**_***A***_***30***_**G**_**29**_**G**_**28**_**G**_**27**_**G**_**26**_					
	**3’G**_**75**_**G**_**74**_**G**_**73**_**G**_**72**_***T***_***71***_***T***_***70***_***T***_***69***_***T***_***68***_***T***_***67***_***G***_***66***_***T***_***65***_***T***_***64***_***T***_***63***_***T***_***62***_***T***_***61***_**G**_**60**_**G**_**59**_**G**_**58**_**G**_**57**_**G**_**56**_***T***_***55***_**G**_**54**_**G**_**53**_**G**_**52**_**G**_**51TFO**_					
**16.** (11-mer) ***Antiparallel***	**5’ C**_**1**_**C**_**2**_**C**_**3**_**C**_**4**_**C**_**5**_**C**_**6**_**C**_**7**_**C**_**8**_**C**_**9**_**C**_**10**_**C**_**11**_	11	0	homopolymer	100	-64.2
	**3’ G**_**22**_**G**_**21**_**G**_**20**_**G**_**19**_**G**_**18**_**G**_**17**_**G**_**16**_**G**_**15**_**G**_**14**_**G**_**13**_**G**_**12**_					
	**5’ G**_**23**_**G**_**24**_**G**_**25**_**G**_**26**_**G**_**27**_**G**_**28**_**G**_**29**_**G**_**30**_**G**_**31**_**G**_**32**_**G**_**33**_**TFO**					
**17.** (11-mer) ***Antiparallel***	**5’ C**_**1**_**C**_**2**_**C**_**3**_**C**_**4**_**C**_**5**_***T***_***6***_**C**_**7**_**C**_**8**_**C**_**9**_**C**_**10**_**C**_**11**_	10/1	1GT 1TG	2 Overlapping	100	-61.4
	**3’ G**_**22**_**G**_**21**_**G**_**20**_**G**_**19**_**G**_**18**_***A***_***17***_**G**_**16**_**G**_**15**_**G**_**14**_**G**_**13**_**G**_**12**_					
	**5’ G**_**23**_**G**_**24**_**G**_**25**_**G**_**26**_**G**_**27**_***T***_***28***_**G**_**29**_**G**_**30**_**G**_**31**_**G**_**32**_**G**_**33**_**TFO**					
**18.** (15-mer) ***Antiparallel***	**5’ C**_**1**_**C**_**2**_**C**_**3**_**C**_**4**_**C**_**5**_**C**_**6**_**C**_**7**_**C**_**8**_***T***_***9***_***T***_***10***_***T***_***11***_***T***_***12***_***T***_***13***_***T***_***14***_***T***_***15***_	8/7	1GT	1 Non-Overlapping	100	-76.4
	**3’ G**_**30**_**G**_**29**_**G**_**28**_**G**_**27**_**G**_**26**_**G**_**25**_**G**_**24**_**G**_**23**_***A***_***22***_***A***_***21***_***A***_***20***_***A***_***19***_***A***_***18***_***A***_***17***_***A***_***16***_					
	**5’ G**_**31**_**G**_**32**_**G**_**33**_**G**_**34**_**G**_**35**_**G**_**36**_**G**_**37**_**G**_**38**_***T***_***39***_***T***_***40***_***T***_***41***_***T***_***42***_***T***_***43***_***T***_***44***_***T***_***45***_**TFO**					
**19.** (15-mer) ***Antiparallel***	**5’ *T***_***1***_***T***_***2***_***T***_***3***_***T***_***4***_***T***_***5***_***T***_***6***_***T***_***7***_**C**_**8**_**C**_**9**_**C**_**10**_**C**_**11**_**C**_**12**_**C**_**13**_**C**_**14**_**C**_**15**_	8/7	1TG	1 Non-Overlapping	100	-82.3
	**3’ *A***_***30***_***A***_***29***_***A***_***28***_***A***_***27***_***A***_***26***_***A***_***25***_***A***_***24***_**G**_**23**_**G**_**22**_**G**_**21**_**G**_**20**_**G**_**19**_**G**_**18**_**G**_**17**_**G**_**16**_					
	**5’ *T***_***31***_***T***_***32***_***T***_***33***_***T***_***34***_***T***_***35***_***T***_***36***_***T***_***37***_**G**_**38**_**G**_**39**_**G**_**40**_**G**_**41**_**G**_**42**_**G**_**43**_**G**_**44**_**G**_**45**_**TFO**					

List of parallel (Sequences 1, 3–15) and antiparallel (Sequences 2, 16–19) DNA triplexes studied in diverse sequential context. The 15-mer triplexes (Sequences 10–14 & 18–19) comprising of 8 G_*_GC & 7 T_*_AT triplets are rearranged to obtain a variety of triplex junctions under different sequence contexts.

Superposition of G_*_GC (green) and T_*_AT (brown) triplets in parallel (**A**) and anti-parallel (**B**) orientation to bring out the nature and source of nonisostericity between triplets. Triplets are depicted as circles formed with the three C1’ atoms of the triplet bases lying on the circumference. Appearance of a twist between the adjacent Hoogsteen bases (G_H_ & T_H_) or reverse Hoogsteen bases (G_RH_ & T_RH_), even prior to the application of formal helical twist (t) constitutes the residual twist Δt°. This together with the radial difference Δr Å between the two superposed triplets, suffice to quantify nonisostericity between them. Note the higher value of Δt° in parallel (**A**) than in antiparallel (**B**) triplets. Illustration of the effect of over winding (30° +Δt°) at the G_H_T_H_ step (**C**) and under winding (30°−Δt°) at the T_H_G_H_ step (**D**) of the Hoogsteen strand consequent to residual twist. Two adjacent G_*_GC triplets with the ideal twist angle of (t) 30° (**E**) is shown to underscore the effect of residual twist.

## Results

### Manifestation of the highest nonisostericity between the parallel G_*_GC & T_*_AT triplets

Superposition of the parallel G_*_GC & T_*_AT base triplets reveals (Fig 1A) misalignment of the C1’ atoms of the Hoogsteen strand (see also S1A Fig). This results in the largest value for the residual twist angle Δt°(± 21.6°) between the G…G and A…T Hoogsteen pairs. It is obvious that the effect of residual twist would be to impart over winding (+Δt°) at the G_H_T_H_ step (G_*_GC preceding T_*_AT) (Fig 1C) and under winding (-Δt°) at the T_H_G_H_ step (T_*_AT preceding G_*_GC) in the Hoogsteen strand of the triplex (Fig 1D). Consequently effective triple helical twist would be 30° (t°) + 21.6° (+Δt°) = 51.6° and 30° (t°)−21.6° (-Δt°) = 8.4° at the GT and TG steps of the Hoogsteen strand respectively. 51.6° is close to nearly twice the value of triple helical twist of 30° (t°), while 8.4° is an exceptionally low value for triple helical twist. Outcome of these creates disconnects of nearly one nucleotide length (5.5 Å to 6.3 Å) in the sugar-phosphate backbone at the successive steps of the Hoogsteen strand (S1B Fig). They also generate severe steric overlap between the adjacent sugars, especially, at the under wound TG step. Bridging these disconnects and ensuring a steric free conformation, while retaining Hoogsteen hydrogen bonds in the G_*_GC & T_*_AT triplets, demand considerable conformational rearrangements (see below). Incidentally, the other nonisostericity parameter viz., radial difference (Δr Å) is relatively small (≅ 0.4 Å) for the parallel (Fig 1A) compared to the antiparallel base triplets (≅ 1.1 Å) (Fig 1B). Given this, it is expected that Δt° would impact more than Δr Å in a parallel DNA triplex.

### Successive incidence of G_*_GC & T_*_AT triplets destabilizes parallel DNA triplex by disrupting Hoogsteen hydrogen bonds

**Fig 2:**
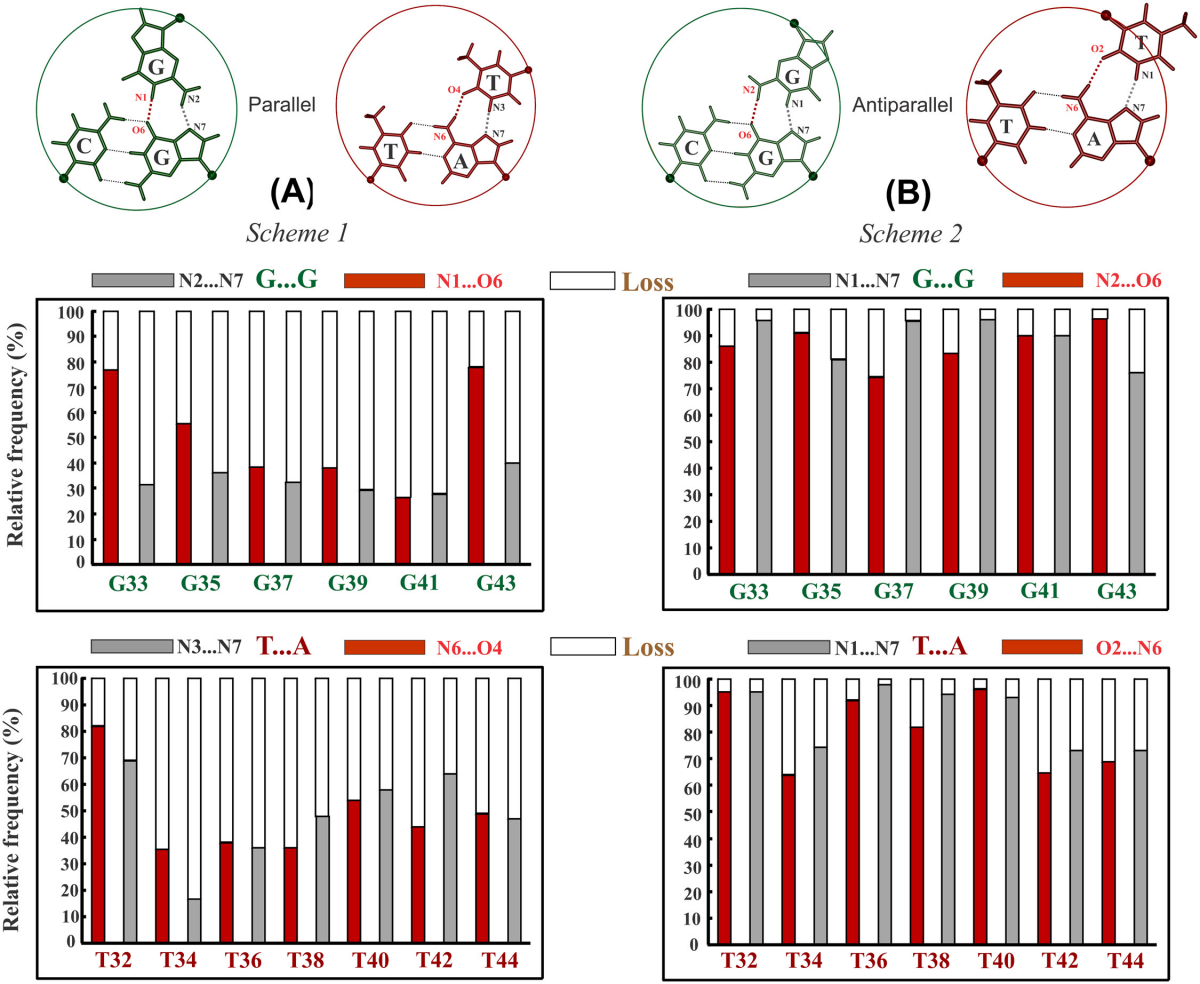
Demonstration of unstable and stable nature of parallel and antiparallel triplex formed by alternating G_*_GC & T_*_AT triplets respectively. **A** Frequency of incidence (red & gray filled part) and loss (void part) of canonical (**A**) Hoogsteen (*Sequence 1*) and (**B**) reverse Hoogsteen hydrogen bonds (*Sequence 2*) in the central 6 G_*_GC and 7 T_*_AT triplets of the 15-mer parallel and antiparallel triplex (terminal triplets not considered) over a simulation time of 250 and 100 ns respectively. Wide spread loss of Hoogsteen hydrogen bonds in the (**A**) parallel triplex (*Sequence 1*), and retention of reverse Hoogsteen hydrogen bonds in the (**B**) antiparallel triplex (*Sequence 2*) are apparent. Canonical Hoogsteen and reverse Hoogsteen hydrogen bonding schemes in the G_*_GC & T_*_AT base triplets in parallel & antiparallel orientations are shown on top for reference.

It might be anticipated that the conformational changes necessitated by the unusually high and low twist angles at the alternating G_H_T_H_ and T_H_G_H_ steps might be absorbed by the inherent flexibility of the triplex. In order to examine this, MD simulations (250 ns) have been carried out on a 15-mer parallel triplex comprising alternating G_*_GC & T_*_AT triplets (Sequence 1 in Table 1). The results at once indicate a proclivity for the loss of Hoogsteen hydrogen bonds in both the triplets (Fig 2A): N6 (A_W_) … O4 (T_H_) and N7 (A_W_) …N3 (T_H_) hydrogen bonds in the T_*_AT triplets are absent for over 52% and 49% of the simulation period respectively, while N7 (G_W_) …N2 (G_H_) and O6 (G_W_)… N1 (G_H_) hydrogen bonds are absent in the G_*_GC triplets for over 64% and 47.7% respectively. This is caused by the movement of T_H_ and G_H_ to overcome the effects of high Δt°. In the process, Hoogsteen hydrogen bonds are disrupted. In striking contrast, the reverse Hoogsteen bonds of the G_*_GC & T_*_AT triplets in the antiparallel DNA triplex (Sequence 2) are largely preserved (Fig 2B). This can be attributed to the much lower value of Δt° (10°) in antiparallel, as opposed to 21.6° in the parallel triplex. Evidently much smaller conformational variations required by the lower residual twists are readily absorbed by the inherent triplex flexibility without the need for the loss of reverse Hoogsteen hydrogen bonds.

#### Emergence of noncanonical Hoogsteen schemes

**Fig 3:**
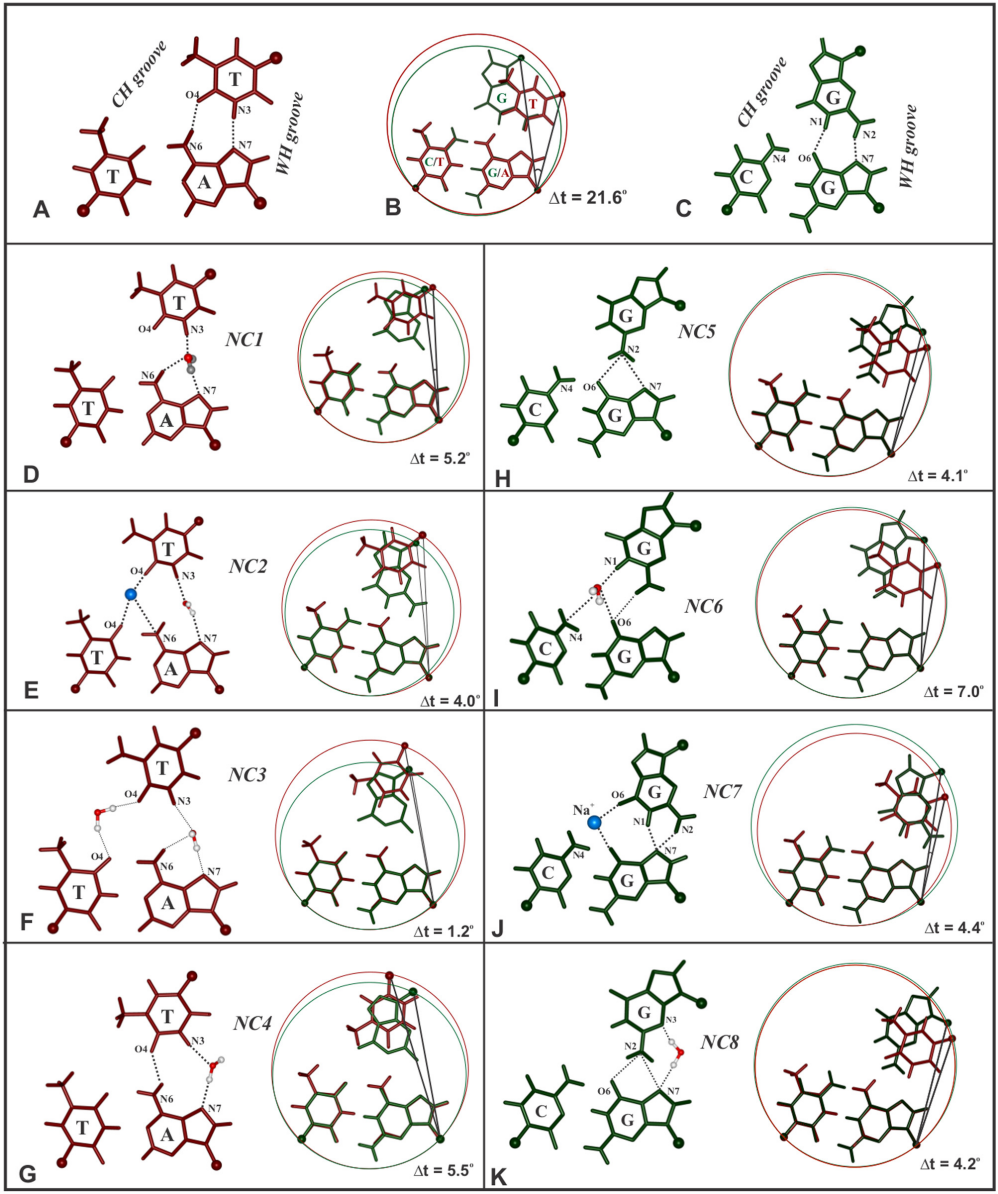
Transition from canonical to noncanonical Hoogsteen hydrogen bond schemes in the T_*_AT (NC1 to NC4) and G_*_GC (NC5 to NC8) triplets with reduced residual twists due to the effects of nonisostericity. Typical canonical Hoogsteen hydrogen bonding schemes in T_*_AT (brown), G_*_GC (green) triplets & their superposition are shown in A, C and B respectively. Noncanonical Hoogsteen hydrogen bonding schemes of T_*_AT (D,E,F,G) & G_*_GC (H,I,J,K) base triplets are shown along with their superposition with the canonical schemes to highlight reduction in the residual twist Δt° (right panel in **D to K**). Na^+^ ion (**E & J**) and oxygen atom of water molecule (**D-G, I & K**) are coloured in cyan and red respectively.

Consequent to the disruption of Hoogsteen hydrogen bonds, alternative schemes of hydrogen bonds emerge for both the T_*_AT (NC1 to NC4; Fig 3D–3G) and G_*_GC (NC5 to NC8; Fig 3H–3K) triplets. These are referred to as noncanonical Hoogsteen schemes. For T_*_AT, NC1 scheme (Fig 3D) is predominant and is formed by way of water mediated interaction involving N3 of T_H_ and N6 & N7 of A_W_. In NC2 (Fig 3E), both ion and water interact with T_H_ (O4 & N3) and N6 & N7 of A_W_ and O4 of T_H_. Interaction via two water molecules describes NC3 (Fig 3F). In all of these none of the canonical Hoogsteen hydrogen bonds are retained. But in NC4 (Fig 3G), canonical N6…O4 Hoogsteen hydrogen bond scheme is preserved while a water molecule mediates the interaction between N3 of T_H_ and N7 of A_W_.

In the case of G_*_GC triplet, NC5 scheme (Fig 3H) is predominant. This is characterised by bifurcated hydrogen bonds involving N2 of G_H_ and O6 & N7 of G_W_. In the NC6 scheme (Fig 3I), a water molecule mediates interaction between G_H_ and WC paired bases. In both these situations, Hoogsteen G_H_ swivels causing the amino group to glide towards the WH groove. But, similar action in the direction of the CH groove results in the NC7 scheme (Fig 3J). Here, an ion comes in between O6’s of G_H_ and G_W_, while N7 of G_W_ forms bifurcated hydrogen bonds with N2 & N1 of G_H_. NC8 scheme is alike NC5, but in addition, is mediated by a water molecule between N7 & N3 atoms of G_W_ and G_H_ (Fig 3K).

A common feature observed in all of the above is the propensity for reducing the initial large Δt° (21.6°) to lower values (as low as 1.2° in NC3; Fig 3G) to circumvent the large mechanistic effects (steric overlap and disconnect in the sugar-phosphate backbone) discussed above. This can happen only at the expense of losing canonical Hoogsteen hydrogen bonding. Such a loss in a large number of triplets would critically affect triplex stability. This is clearly reflected in the very low binding free energy of only—9.4 kcal/mol for the parallel triplex (Sequence 1) in sharp contrast to the comparatively high value of—70.80 kcal/mol for the antiparallel triplex (Sequence 2). The latter by and large retains all the reverse Hoogsteen hydrogen bonds in all the triplets. Thus, the parallel DNA triplex with consecutive G_*_GC and T_*_AT triplets is not expected to be stable due to the mechanistic effects of extreme nonisostericity at every base step of the Hoogsteen strand. Hence the discussion about this unstable structure is not pursued further.

### Limited number of nonisosteric base triplets (NIBTs) does not impinge on triplex stability

In the 15-mer parallel DNA triplex (Sequence 1, Table 1) discussed above, each triplet say T_*_AT, is flanked on either side by the nonisosteric base triplet G_*_GC, giving rise to two pairs of consecutive and overlapping nonisosteric base triplets (NIBTs), G_*_GC & T_*_AT and T_*_AT & G_*_GC. The intervening T_*_AT triplet, being common to both, overlaps with both the flanking G_*_GC triplets, and it therefore in effect experiences nonisostericity effects due to the preceding and the following G_*_GC base triplet. Consequently, two Δt°s, viz., +Δt° at the GT step (between the G_*_GC & T_*_AT triplets) and−Δt° at the TG step (between the T_*_AT & G_*_GC triplets) (Fig 1C) operate on the intervening T_*_AT triplet. We refer to this scheme as overlapping pair of triplets. In fact, the 15-mer triplex (Sequence 1; Table 1) may deem to be comprised of such trimer triplets constituting 14 pairs of overlapping NIBTs. The deleterious effect of which has been discussed above. But in reality a limited number of NIBTs may occur in the target duplex. Moreover, these may be spaced out rather than occurring alternately along the sequences. Such circumstances are in fact investigated [79,80]. These prompted us to study the effect of isolated and lone occurrence of such NIBTs. This situation can be visualized when a G_*_GC or T_*_AT triplet interrupts an otherwise homopolymeric triplex (Sequences 4 and 8; Table 1). It will be intriguing to examine if the Hoogsteen hydrogen bonding pair is retained here and if not, evaluate its influence on the triplex structure and stability.

#### Structure of a T*AT triplex with a G*GC interruption mediated by overlapping pairs of NIBTs (-Δt° followed by +Δt°)

**Fig 4:**
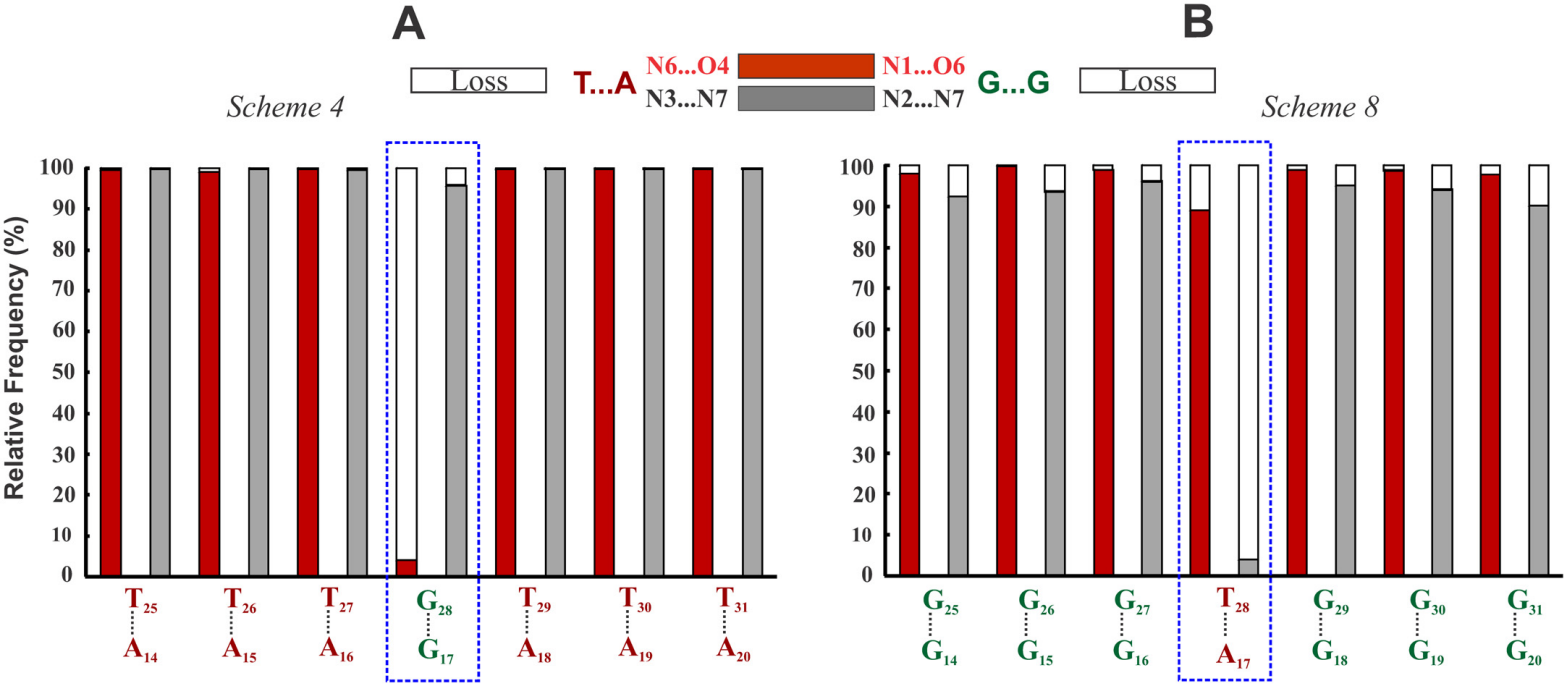
Destabilization of canonical Hoogsteen hydrogen bond in the G_*_GC and T_*_AT interruptions. Frequency of incidence (red & gray filled part) and loss (void part) of Hoogsteen hydrogen bonds in the G_*_GC interruption of the T_*_AT triplex (**A**) (*Sequence 4*) and in the T_*_AT interruption of the G_*_GC triplex (**B**) (*Sequence 8*). Loss of the canonical Hoogsteen hydrogen bond (void part) in the interrupting G_*_GC (N1…O6) and T_*_AT (N3…N7) triplets are conspicuous (blue box).

**Fig 5:**
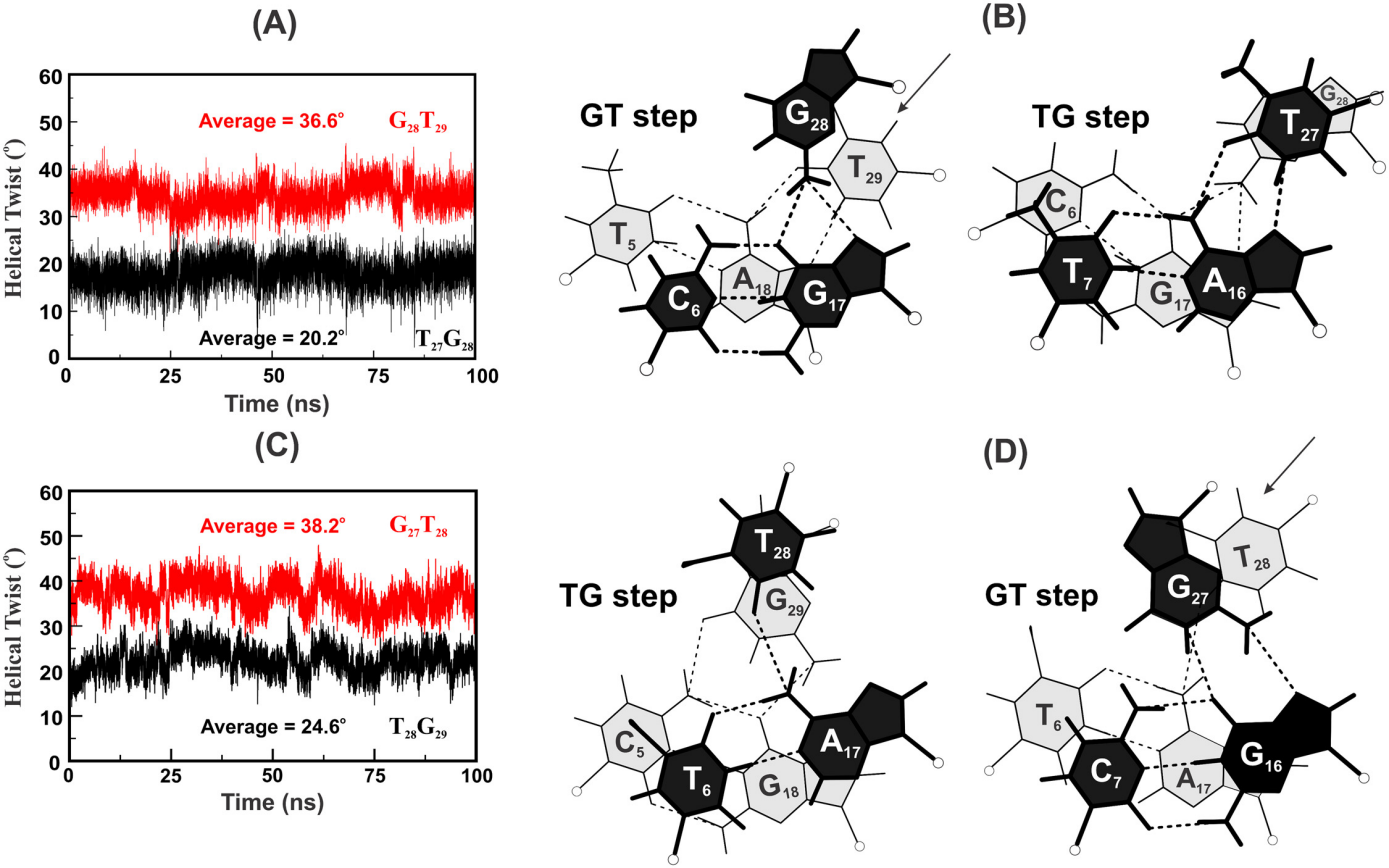
High and low twist at the GT & TG steps concomitant with disrupted stacking at the interruption sites. Helical twist angle variation at the overlapping GT and TG steps at the G_*_GC interruption site (**A**) (*Sequence 4*) in the T_*_AT triplex and in the G_*_GC triplex (*Sequence 8*) with the T_*_AT interruption (**C**). Note the loss of significant base stacking at the GT step of the interruption site (**B**) and (**D**) and partial stacking at the TC & CT steps of WC duplex.

It is readily seen that a G_*_GC triplet interruption in the parallel homopolymeric T_*_AT DNA triplex (Sequence 4; Table 1) generates two sets of NIBTs which are overlapping and consecutive. The first pair of NIBT at the TG (T_27_G_28_) step results in a residual twist of -Δt° =−21.6° and effects under winding. This is followed by a residual twist of +Δt° = +21.6° at the GT (G_28_T_29_) step to effect over winding. MD simulation of this triplex reveals that the interrupting G_28*_G_17_C_6_ triplet loses the N1…O6 hydrogen bond of the canonical Hoogsteen scheme (Fig 4A and S2B Fig) in favour of the noncanonical NC5 Hoogsteen scheme (Fig 3H). This clearly demonstrates that even an isolated nonisosteric triplet is unable to retain the canonical Hoogsteen pair and instantly transits to a noncanonical scheme (NC5) which reduces the Δt° values to ~−4.1° and + 4.6° at the T_27_G_28_ and G_28_T_29_ steps respectively from the initial value of 21.6°. As a result, slightly lower (20.2°) and higher (36.6°) helical twist angles are seen at these steps occurring on either sides of the G_*_GC interruption (Fig 5A). Stacking interactions between the Hoogsteen bases (G_28_ and T_29_) are minimal at the GT step compared to that between T_27_ & G_28_ at the under wound TG step (Fig 5B). Better stacking between A_16_ & G_17_ and G_17_ & A_18_ and relatively poor stacking between T_5_ & C_6_ (Fig 5B) of WC paired duplex prevail. Twist angles at the TT Hoogsteen steps and the TC & CT steps of WC duplex lie around 29.1°.

Average value for the X-displacement of base pair at the interruption site (-3.6 Å) is slightly higher compared to other places (-2.9 Å). On the other hand, propeller twist of WC base pairs at the interruption site is slightly lower (~ 6.86°) compared to -9.6° found at other places. Sugar ring of G_28_ of the G_28*_G_17_C_6_ interruption assumes the C1’ *exo* pucker, while thymidines of the Hoogsteen strand favour O4’ *endo* pucker. All the torsion angles lie in their preferred ranges. Average value of minor groove width is lowered to 12.2 Å from the initial value of 14 Å concomitant with an increase in the major groove width by 1.5 Å to a value of 20.1 Å. Increase in the widths of WH and CH groove by 2 and 4 Å to 9.3 Å and 16.1 Å respectively is also seen (S3A Fig). The binding free energy (~ -53.5 kcal/mol) of this triplex (Sequence 4) is reduced by ~ 10 kcal/mol compared to ~ -63.6 kcal/mol obtained for the uninterrupted homopolymeric T_*_AT triplex (Sequence 3; S2A Fig) pointing clearly to the destabilizing effect of base triplet interruption which is tolerated as a triplet mismatch. Incidentally, a T_*_AT triplex with a G_*_GC interruption forms a stable parallel triple helix [62].

#### Structure of a G*GC triplex with a T*AT interruption mediated by overlapping pairs of NIBTs (+Δt° followed by -Δt°)

Here, the roles of G_*_GC and T_*_AT triplets are swapped. A homopolymeric G_*_GC triplex is interrupted by the T_28*_A_17_T_6_ triplet, creating two overlapping and consecutive pairs of NIBTs (Sequence 8; Table 1). Contrary to the previous case, here the over winding GT step (+Δt°) precedes the under winding TG step (-Δt°) in the Hoogsteen strand. MD simulations show that the interrupting T_28*_A_17_T_6_ loses the canonical Hoogsteen N3 (T_H_)…N7 (Aw) hydrogen bond for over 95% of total simulation time (Fig 4B and S4B Fig), while the O4 (T_28_)…N6 (A_17_) hydrogen bond is lost for over 13% of total simulation time. Eventually, a water mediated noncanonical NC1 Hoogsteen scheme (Fig 3D) prevails, as a triplet mismatch, entailing reduced Δt° values of +5.8° and -6.1° at the Hoogsteen GT and TG steps respectively. Akin to the previous case (Sequence 4; Table 1), twist angles (Fig 5C), base stacking pattern (Fig 5D), groove width variations (S3B Fig) and base pair parameters exhibit variations at the interruption site similar to above. Average value of minor groove width is ~ 13.26 Å with a reduced value of ~12.1 Å at the interruption site (S3B Fig). Concomitantly, major groove widens by ~2.5 Å to a value of ~ 21.9 Å. Likewise, CH groove widens by ~ 1.5 Å to ~ 12.3 Å with the WH groove remaining nearly unchanged at ~ 9.9 Å (S3B Fig). The binding free energy of this triplex with an interruption is reduced by ~ 11.4 kcal/mol to ~ -50.6 kcal/mol compared to ~-62.0 kcal/mol obtained for the uninterrupted homopolymeric G_*_GC triplex (Sequence 7; S4A Fig).

On the other hand, MD simulation of an 11-mer antiparallel G_*_GC triplex with (Sequence 17) and without a T_*_AT triplet interruption (Sequence 16) entails only a marginal loss of ~ 3 kcal/mol in the binding free energy due to the retention of canonical reverse Hoogsteen hydrogen bonds even in the T_*_AT interrupt (S5 Fig).

#### Multiple interruptions progressively destabilize the triplex

To envisage further the effect of multiple interruptions (NIBTs), a parallel T_*_AT DNA triplex with 2 and 3 G_*_GC interruptions, separated by 3 and 2 T_*_AT triplets (Sequence 5 and Sequence 6), are investigated by MD simulation. Expectedly, the canonical Hoogsteen hydrogen bonds are lost in all the G_*_GC interruptions, G_15_…G_26_ & G_19_…G_30_ in sequence 5 and in G_14_…G_25_, G_17_…G_28_ & G_20_…G_31_ in sequence 6 (S2C and S2D Fig) and figure as triplet mismatches. Other conformational changes are similar to those seen with only one interruption (Sequence 4). The binding free energy of the triplex with 2 and 3 G_*_GC interruptions (Sequence 5;−40.2 kcal/mol and Sequence 6;−25.9 kcal/mol; Table 1) is reduced further by ~13 and 27 kcal/mol respectively compared to the triplex with a single interruption (Sequence 4) indicating progressive destabilization of triplex with increasing number of interruptions. Likewise, 2 T_*_AT interruptions in the parallel G_*_GC DNA triplex (Sequence 9) lose the canonical Hoogsteen hydrogen bonds involving A_15_…T_26_ & A_19_…T_30_ pair (S4C Fig) concomitant with binding energy loss of ~−11 kcal/mol compared to the triplex with a single T_*_AT interruption (Sequence 8 vs. Sequence 9). These indicate progressive destablisation of triplex with increasing number of interruptions with a near linear correlation (S6 Fig). This could impact triplex stability or even its formation. While longer sequences might be able to endure interruptions, shorter sequences with frequent interruptions may fail to form triplex.

### Influence of a solitary pair of NIBTs on triplex stability

In view of the above findings it is extremely important to ascertain the influence of a single pair vis-à-vis 2 overlapping pairs of NIBTs in retaining or disruption of the canonical Hoogsteen hydrogen bonds and their impact on triplex stability. A single pair of (non-overlapping) NIBTs is harbored uniquely at a triplex junction interface. This can be differentiated by an over winding GT (+Δt°) or an under winding TG (-Δt°) step along the Hoogsteen strand. Results of MD simulations of 5 DNA triplexes differing in the number of triplex junctions and GT/TG step junction interfaces (Sequences 10–14; Table 1), mimicking a few experimentally studied instances [72, 76, 80, 81] are discussed below.

#### Structure of the DNA triplex junction with a GT step (+Δt°) interface

**Fig 6:**
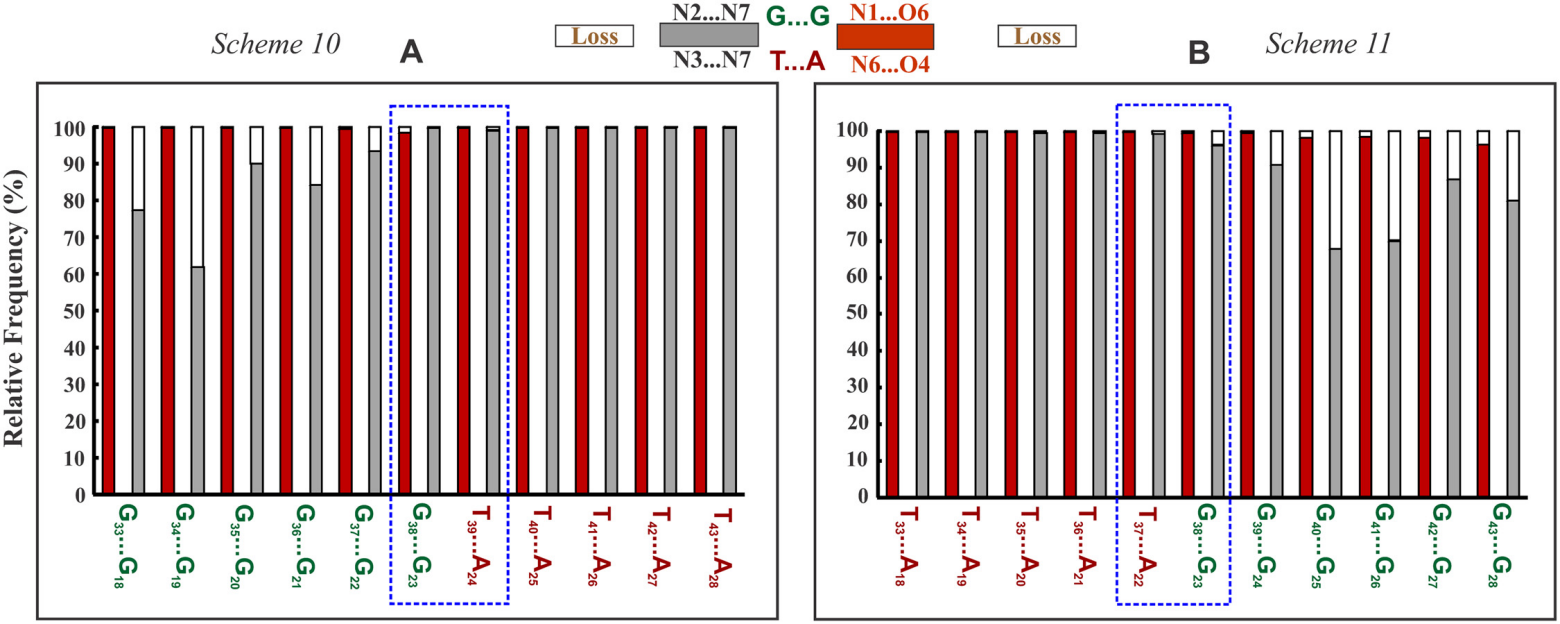
Demonstration of the retention of Canonical Hoogsteen hydrogen bonds at the G_*_GC/T_*_AT triplex junction interface. Frequency of incidence (red & gray colour part) and loss (void part) of Hoogsteen hydrogen bonds in the NIBTs at the GT step—*Sequence 10* (**A**) and at the TG step—*Sequence 11* (**B**) triplex junction interfaces. Hydrogen bonds retention is highlighted by the enclosed blue box.

**Fig 7:**
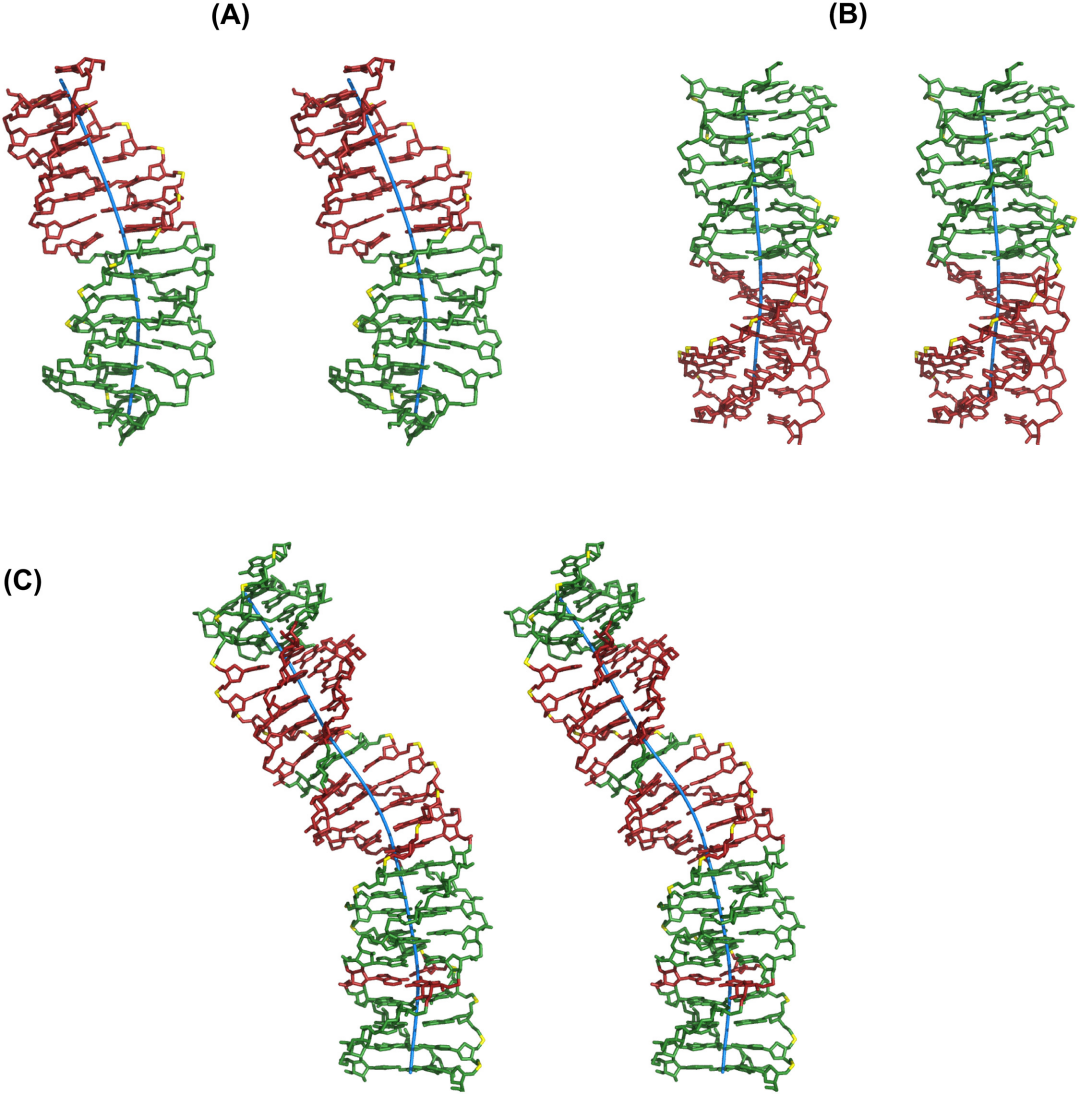
TFOs leading to an overwinding GT step triplex junction interface act as bending agent. Stereo view of parallel triplex with (**A**) a GT step junction interface (Sequence 10), (**B**) a TG step junction interface (Sequence 11) and (**C**) a triplex containing a GT and a TG step junction interface as well as interruptions (1 T_*_AT & 1 G_*_GC) (Sequence 15). Note a conspicuous bend in the triplex structure with GT step junction interface in the sequences 10 & 15 (**A** & **C**), while near uniform triplex structure prevails in the sequence 11 with a TG step junction interface (**B**). G_*_GC and T_*_AT triplets are coloured green and red respectively. Helical axis (blue stick) w.r.t WC duplex is shown. Phosphate atom of the third strand (TFO) is coloured yellow for reference.

Sequence 10 in Table 1 represents a mini 8-mer G_*_GC triplex followed by a mini 7-mer T_*_AT triplex constituting a triplex junction harboring a lone pair of NIBTs mediated by the over winding G_38_T_39_ step (+Δt°). MD simulation of this structure surprisingly shows the retention of canonical Hoogsteen hydrogen bonds (Fig 6A) in both the NIBTs. Effect of overwinding at the GT step (+Δt°) is reflected by way of large X-displacement of WC pairs in the junction neighborhood (S7A Fig) viz., C_8_…G_23_ exhibiting larger value (-9.23 Å) followed by C_9_…G_22_ (-4.7 Å) and C_10_…G_21_ (-3.7 Å). X-displacement is around -3.4 Å and -3.1 Å for the other C…G pairs (in G_*_GC triplex) and T…A pairs (in T_*_AT triplex) respectively. As expected, higher twist angle of ~ 37.5° at the G_38_T_39_ step of the junction interface concomitant with a low twist of ~ 20° prevails at the T_7_C_8_ step of WC duplex (S8A Fig). These cause a conspicuous bend in the triplex (Fig 7A). Twist angles at the TT and CC steps of the WC duplex fluctuate around 29.1°, while propeller twists of C…G and T…A pairs remain around -1.3° and -6.33° respectively. Stacking interaction is affected in the neighborhood of junction interface with minimal stacking between the G_37_ & G_38_ and T_39_ & T_40_ of the Hoogsteen strand, while good stacking prevails between the adjacent triplets in the rest of triplex (S8C Fig). Both major and minor groove expand by ~ 2.5 Å and ~ 1 Å to settle around ~ 23.71 Å and 13.8 Å respectively. WH groove of the mini T_*_AT triplex widens from 7.3 Å to 9.2 Å with negligible change in mini G_*_GC triplex (~ 9.0 Å). CH groove of the T_*_AT mini triplex widens to ~18 Å, while that of G_*_GC mini triplex narrows to ~ 12.0 Å starting from ~14 Å respectively (S3C Fig). Backbone torsion angles lie in the preferred range (S9A Fig). The binding free energy of this triplex is ~ -71.6 kcal/mol.

#### Structure of the triplex junction harboring the TG step (-Δt°) interface

Sequence 11 in Table 1 corresponds to a triplex junction with a single pair of NIBTs intervened by the T_37_G_38_ step. Here again the results of MD simulation reveal preservation of canonical Hoogsteen hydrogen bonds in the nonisosteric triplet pairs (Fig 6B). The under winding effect caused by the T_37_G_38_ step is reflected in the low X-displacement for the WC pairs, C_8_ …G_23_ (~ -1.31 Å) and T_9_…A_22_ (~ -0.65 Å) respectively, proximal to the triplex junction. This is in contrast to large X-displacement seen at the GT junction interface (see above). X-displacement for the other C…G and T…A pairs correspond to ~ -3.23 Å and -2.45 Å respectively. Although a small warp is observed at the triplex junction (Fig 7B) it is not as prominent as in GT step junction interface (Sequence 10). Interestingly BII phosphodiester conformation is found at the under wound T_37_G_38_ (S9B Fig) while the rest favour BI. As expected, a low twist angle of ~ 15° is found at the Hoogsteen T_37_G_38_ step concomitant with a higher twist at the abutting C_8_T_9_ (~38.3°) and C_7_C_8_ (~35.1°) steps of WC duplex (S8B Fig). Twist angles at the other CC & TT steps of the WC duplex remain at ~ 30°. As in the previous case, propeller twist for the C…G pair (-4.02°) is lower than T…A pair (-13.7°). Average minor groove width is ~13.12 Å in the mini G_*_GC triplex and it is ~11.7 Å in the mini T_*_AT triplex. Average major groove width in both the triplexes is ~ 21.1 Å. Likewise, WH and CH groove width lie in the range of ~ 9 Å and 13 Å respectively (S3D Fig). Stacking interaction is minimal at the neighborhood of triplex junction (S8D Fig) involving Hoogsteen bases, G_38_ & G_39_ and WC duplex pyrimidines, C_7_ and C_8_. However partial stacking prevails between the purines, A_22_ & G_23_ of WC duplex Binding free energy of this triplex corresponds to ~ -78.4 kcal/mol. These data clearly brings about the distinctions in the influence of overlapping and non-overlapping NIBTs.

#### Incidence of multiple junctions contributes to instability factor

MD simulations of 15-mer triplexes with the incidence of two (Sequences 12–13; Table 1) and four (Sequence 14; Table 1) triplex junctions under different sequence contexts are performed to discern the limit on the number of occurrences of non-overlapping pair of NIBTs that might sustain, hinder or completely prevent triplex formation. In sequence 12, the 8-mer G_*_GC triplex is flanked by T_*_AT triplexes creating a T_34_G_35_ step junction interface on the 5’-side and G_42_T_43_ step junction interface on the 3’-side of TFO. On the other hand, in sequence 13, the 7 mer T_*_AT triplex is flanked by G_*_GC triplexes creating a G_34_T_35_ junction interface on the 5’-side and a T_41_G_42_ step interface on the 3’-side of TFO. Both these triplexes possess two sets of non-overlapping pair of NIBTs spaced apart by at least 7 or more triplets. sequence 14 is designed to contain 4 sets of non-overlapping NIBTs to examine the effect of increased incidence of triplex junctions. Results reveal canonical Hoogsteen hydrogen bonds are retained at the junction NIBTs akin to sequences 10 & 11 with only junction. Features characteristic to the GT or TG step junction interface including conspicuous bend at the over wound Hoogsteen GT (+Δt°) steps (G_42_T_43_ in Sequence 12, G_34_T_35_ in Sequence 13 and G_36_T_37_ in Sequence 14; S10A–S10C Fig) are manifested here as well. The 15-mer triplexes with 2 junctions or 2 sets of non-overlapping pairs of NIBTs) have binding free energies of ~ -57.2 kcal/mol (Sequence 12) and ~ -55.8 kcal/mol (Sequence 13). The 15-mer triplex with 4 junctions or 4 sets of non-overlapping pairs of NIBTs shows a further decrease in binding free energy of ~ -44.3 kcal/mol (Sequence 14) reflecting the progressively declining stability of triplexes with increase in number of non-overlapping pairs of NIBTs as well (S11 Fig). This clearly indicates that although not as detrimental as the overlapping NIBTs incidence of large non-overlapping NIBTs in a given sequence could affect triplex stability.

### Structure of a triplex with simultaneous incidence of junctions and interruptions

Here, we have investigated the impact of 2 interruptions (4 pairs of overlapping NIBTs) and 2 junctions (2 pairs of non-overlapping NIBTs), which together makeup six pairs of NIBTs (~ 25%) in the 25-mer triplex (Sequence 15; Table 1). Results are on the expected lines with disengagement of canonical Hoogsteen hydrogen bonds to noncanonical Hoogsteen sequences in the T_55*_A_30_T_21_ and G_66*_G_41_C_10_ interruptions as in the sequences 4 to 9, while they are retained in at the triplex junction interfaces G_60*_G_35_C_16_ & T_61*_A_36_T_15_ and T_71*_A_46_T_5_ & G_72*_G_47_C_4_ triplets as in the sequences 10–14. Likewise, trend of high (~37°) and low twist (~12.7°) angles at the Hoogsteen G_60_T_61_ and T_71_G_72_ steps respectively concomitant with the reverse trend at the corresponding steps of WC duplex namely; low (~20.6°) and high (~37.2°) twist angles at the T_15_C_16_ and C_4_T_5_ steps respectively, are noticed. Also, larger X-displacement (-7.6 Å) is seen for the C_17_…G_34_ and C_18_…G_33_ WC base pairs causing a bend in the triplex (Fig 7C). This triplex with 6 pairs of NIBTs is associated with a binding free energy of ~ -81.2 kcal/mol.

## Discussion

Ever since the finding of the ability of nucleic acid duplex to accommodate a TFO along its major groove, a variety of biological roles have been demonstrated for DNA, RNA and DNA.RNA hybrid triplexes. Distinctive disposition of donors and acceptors in guanine and thymine of the GT rich TFOs enables them to interact with the purine rich strands of WC duplexes in both parallel and antiparallel orientations forming G_*_GC and T_*_AT triplets, with unique geometries rendering them to be nonisosteric. Knowledge of the nature and magnitude of nonisostericity between triplet pairs is crucial in the design of TFOs as they determine the degree of influence on triplex conformation as well as stability. Nonisostericity can be effectively and elegantly defined by residual twist and radial difference [82] which can relate directly to structural traits. More assuring is that they have proven to be valuable in assessing the impact of nonisostericity on DNA triplexes [82, 83].

Surprisingly G_*_GC & T_*_AT parallel triplets exhibit strikingly large nonisostericity with the highest value of ~21.6° for the residual twist (Δt°) making it a potential culprit for the observed selective preference of parallel triplexes [73, 74, 75, 77] as this is expected to strongly perturb triplex. Qualitative arguments based on nonisostericity are unable to offer clarity to explicate the above, leave alone a mechanistic rationale. MD simulations of 14 parallel and 5 antiparallel DNA triplexes (Table 1), comprising these triplets in an assortment of sequence contexts, are carried out to critically evaluate the decisive influence of single, double and multiple pairs of overlapping and non-overlapping nonisosteric base triplets (NIBTs) on triplex stability. Results have provided new insights for a comprehensive understanding of nonisostericity effects which should aid in the efficient design of TFO to target nucleic acids duplexes to form triplexes.

### Relative influence of residual twist vis-à-vis radial difference

One of the key revelations from the present study is the disruption of canonical Hoogsteen hydrogen bonds in both the G_*_GC and T_*_AT parallel triplets when they occur alternately (Sequence 1; Fig 2A) and as interruptions (Sequences 4–6, 8, 9; Fig 4A and 4B & S2 and S4 Figs) in a parallel triplex in sharp contrast to antiparallel triplex where reverse Hoogsteen hydrogen bonds remain intact (Fig 2B and S5 Fig). This is directly correlatable to the vastly contrasting nonisostericity between the triplet pairs in parallel and antiparallel orientations, as reflected in the disparate values of Δt° and Δr Å. While Δt° is twice (21.6°) the value in parallel compared to 10.6° in antiparallel triplexes, Δr Å is slightly larger in antiparallel (1.1 Å) than in the parallel (0.4 Å) triplexes. Though both Δt° and Δr Å independently impact in creating backbone disconnect at the successive phosphodiester links, influence of Δr Å seems to be more in the antiparallel triplex although the effects are absorbed by the intrinsic triplex flexibility. On the other hand, mechanistic influence of the large residual twist of 21.6° in parallel triplexes is reflected in lowering the stability sharply through disruption of Hoogsteen hydrogen bonds and associated effects through significant conformational modifications (Figs 2A, 3, 4 & 7). In contrast, smaller Δt° (~10.6°) merely causes a zig-zag sugar-phosphate-sugar backbone conformation concomitant with minor twist angle variations in the antiparallel triplex and these by no means affect its formation [83]. Therefore, it can be reckoned that the role of Δt° is more dominant than Δr Å in influencing the formation as well as stability of triplexes.

### Overlapping NIBTs disrupt canonical Hoogsteen hydrogen bond and stacking

**Fig 8:**
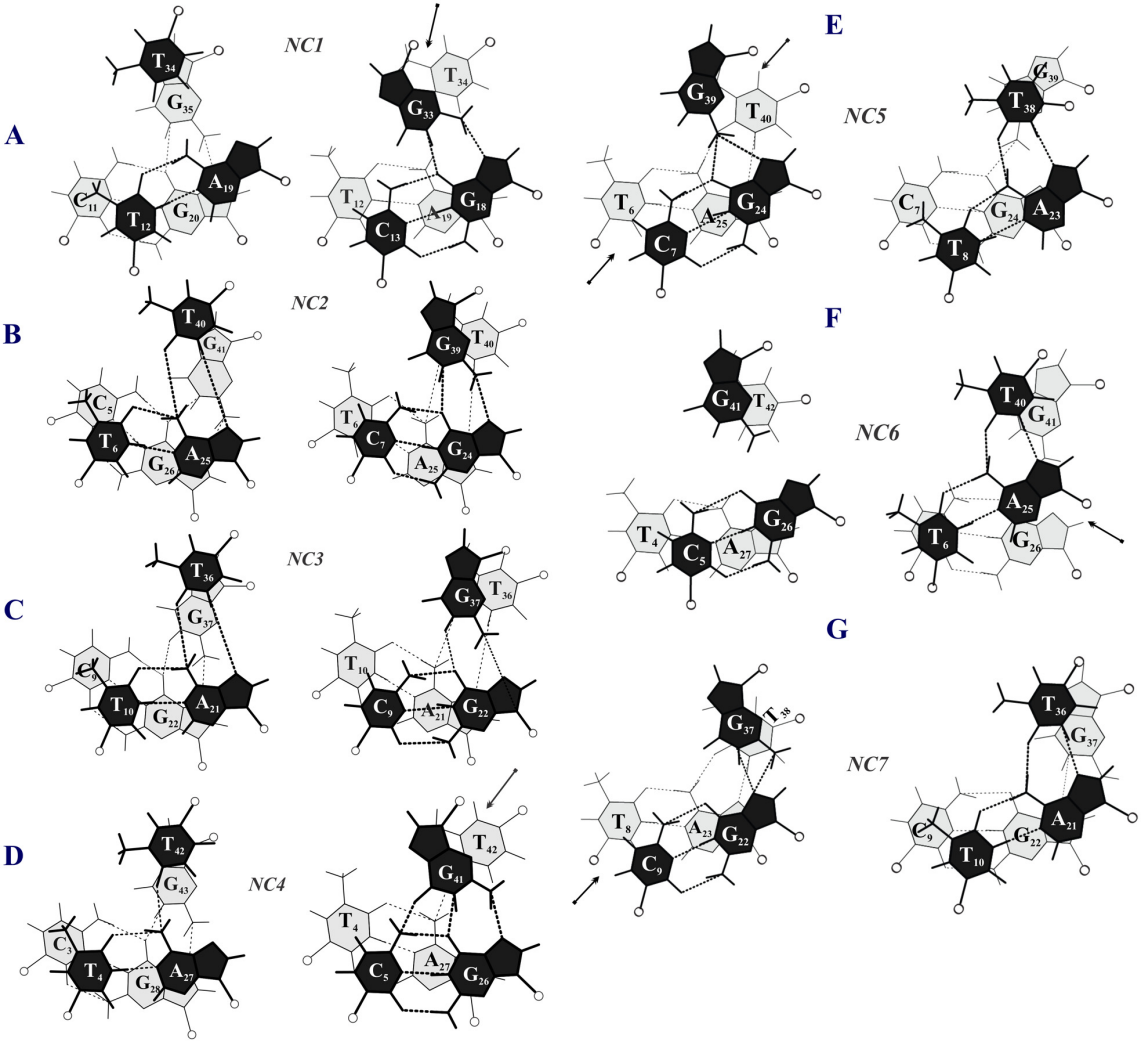
Incidence of alternating G_*_GC and T_*_AT triplets in a parallel triplex disrupts base stacking. Stacking interaction at the GT and TG steps of the Hoogsteen strand in the various noncanonical Hoogsteen schemes seen for the *sequence* 1 during simulation: NC1 to NC4 for T_*_AT and NC5 to NC7 for G_*_GC triplets. Loss of stacking is indicated by dark arrows. C1’ atom of the sugar is shown as open circle.

Isosteric base triplets (with inherent Δt° = 0 and Δr Å = 0) endow a ‘uniform’ DNA triplex. However, presence of residual twist (Δt°), as large as 21.6° between the parallel G_*_GC & T_*_AT triplets undermines the cannonical Hoogsteen hydrogen bonds in the DNA triplex causing them to assume noncanonical Hoogsteen schemes (NC1 to NC4 for T…A and NC5 to NC8 for G…G; Fig 3). By doing so, nonisosteric effects are offset via reduction in Δt° (Fig 3D–3J) concomitant with loss of adjacent base stacking (Fig 8). Thus, loss of canonical Hoogsteen G…G and T…A hydrogen bonds together with interrupted stacking decrease triplex stability. Absence of these in the antiparallel DNA triplex [82] results in highly favorable binding free energy of -70.8 kcal/mol compared to -9.4 kcal/mol for the parallel triplex. It is evident then that parallel triplexes with frequent juxtaposition of G_*_GC and T_*_AT triplets would be less favored.

### Comparative assessment of overlapping vis-à-vis non-overlapping NIBTs on structure, energetics and stability of triplexes

As mentioned earlier, a single base triplet interruption in an otherwise homopolymeric triplex (typified by isosteric base triplets) results in two consecutive and overlapping pairs of NIBTs, with the interrupting NIBT simultaneously bearing the brunt of nonisostericity effects of +Δt (+21.6°) and -Δt (-21.6°) (Sequences 4,8; Table 1). This causes the canonical Hoogsteen hydrogen bond to switch to noncanonical scheme (Fig 3D and 3H), resulting in disrupted base stacking (Fig 5B and 5D). It is obvious that loss of even one of the canonical hydrogen bonds in the triplet interruption enables the Hoogsteen base to sway in a direction to elude the deleterious effect of large Δt°. The base triplet interruption under these circumstances is reckoned as a triplet mismatch and a limited number of them, spaced apart, could be tolerated in a sufficiently long sequence (Sequence 15). Systematic investigations made here suggests that an interrupting G_*_GC or T_*_AT triplet decreases the binding energy by nearly 10–11 kcal/mol with reference to the homopolymeric triplex (Sequence 3 vs. Sequence 4 and Sequence 7 vs. Sequence 8). This reduction nearly doubles to ~ 23 kcal/mol with 2 interruptions (Sequence 3 vs. Sequence 5 and Sequence 7 vs. Sequence 9, Table 1). Increase in the incidence of interruptions from 1 to 3 (Sequence 4 vs. Sequence 6) further reduces the binding energy by ~ 37 kcal/mol indicating a linear correlation between the base triplet interruptions and the binding energy penalty. Hence, it is obvious that multiple incidence of interruptions ie. overlapping pair of NIBTs leads to less stable nature of parallel triplex leading even to inhibition of triplex formation. On the other hand, in the antiparallel triplex (Sequence 16 vs. Sequence 17), the estimated loss in the binding energy (~3 kcal/mol) caused by an interruption due to retention of canonical reverse Hoogsteen hydrogen bond (S5 Fig) and marginal loss in stacking is not severe or detrimental to antiparallel triplex formation.

On the other hand incidence of non-overlapping pairs of NIBTs harboring at triplex junctions (Sequences 10–14) experiences nonisostericity effects of either +Δt° or -Δt° alone. The over winding influence of +Δt° at the GT step causes the triplex to bend (Fig 7A and 7C and S10 Fig). Unwinding influence of -Δt° at the TG step merely brings about change from BI to BII phosphodiester conformation (S9 Fig; Sequences 11–14). Comparison of the binding energies of the five 15-mer triplexes (Sequences 10 to 14) designed out of 8 G_*_GCs and 7 T_*_ATs suggest a reduction in the binding energy with the increase from 2 (Sequences 12 and 13) to 4 (Sequence 14) in the number of non-overlapping NIBTs (triplex junctions). Sequences 12 and 13 have 1 TG and 1 GT junction while in Sequence 14 there are 2 TG and 2 GT junctions. This causes a reduction in binding energy of ~ 11.5 to 13 kcal/mol (Sequence 13 & Sequence 14 and Sequence 12 & Sequence 14) when non-overlapping NIBTs are increased from 2 to 4. Using this, a crude estimate of the binding energy penalty for a non-overlapping pair of NIBTs turns out to be ~ 5.8 to 6.5 kcal/mol. This is nearly one half compared to the binding energy penalty (~ 11 to 14 kcal/mol) for an interruption entailing 2 overlapping pairs of NIBTs.

Antiparallel triplexes with GT or TG junction interfaces (non-overlapping pair of NIBTs) (Sequence 18 & Sequence 19) retain canonical reverse Hoogsteen hydrogen bonds (data not shown) at the junction interface like in parallel triplexes. Their binding energies of -76.4 kcal/mol (Sequence 18) and -82.3 kcal/mol (Sequence 19) are slightly more favorable by ~ 5 kcal/mol than their parallel counterparts (-71.6 kcal/mol and -76.4 kcal/mol) due to better stacking at the interface.

### Bent triplex as a possible recognition feature for triplex binding proteins

It is significant that characteristic bending of ~ 25° (Fig 7A and 7C and S10 Fig) is seen in the GT step triplex junction interfaces (Sequences 10, 12–14). Also, it has been shown earlier [83] that the GA step triplex junction interface also exhibits bending of ~ 17°. Besides, it is known that a tethered GT rich TFO induces a bend in the triplex formed [84, 85] which regulates the transcription of Luciferase gene [86]. Therefore it might be speculated that bent triplexes could serve as recognition sites for triplex binding proteins. Interestingly, an intramolecular triplex formed by GT rich TFO is demonstrated to bind a few hnRNP family proteins [87]. Likewise an intermolecular triplex formed by a CT rich TFO is shown to bind the GAGA transcription factor from drosophila melanogoster [88].

### Parallel between the number and variety of NIBTs and experimental data

Present results convincingly provide a mechanistic argument for the experimental observations concerning the ability or lack of it for formation of parallel DNA triplex with G_*_GC & T_*_AT triplets and preference of antiparallel over triplex. For instance the 22-mer TFO, GG*GT*T**GT**GG*GT*T**GT**GGGGG**T**GG targeted to form parallel triplex with the human Ki-*ras* promoter [77], entails 5 interruptions (marked bold) resulting in 8 sets of overlapping pairs of NIBTs, of which 3 occur consecutively twice in the sequence. Apart from this, 2 sets of non-overlapping pairs of NIBTs (mini triplex junctions marked in *italics* and underlined) occur in the sequence. As a consequence, it is expected that a parallel triplex would be less stable owing to the disruption of canonical Hoogsteen hydrogen bonds (*5 out of 22*) in the 5 triplet interruptions (3 T_*_AT & 2 G_*_GC). Indeed, this TFO forms only antiparallel triplex. Similarly, the 31-mer TFO, 5’*GT*TTT*TG*G**GTG**T**TGTG**GGTGTGTGTGGTT*TG*, designed to inhibit transcription of HIV-1 in infected human cells [75] could bind target duplex only in anti-parallel and not in parallel orientation due to presence of 12 sets of overlapping pairs of NIBTs due to 8 interruptions, spaced in proximity, effecting disruption of Hoogsteen hydrogen bonding in 8 out of 31 triplets. On the other hand, a G_*_GC triplet interruption in 5’TTT**TGT**TTTG [62] and 5’TTCTTCT**TGT**TTCT [89] does not inhibit parallel triplex formation due to loss of only one Hoogsteen hydrogen bond out of 9. Likewise the 14-mer TFO, 5’ **GTGGTG**G**GTGTGTG** can form only antiparallel triplex [73] since in parallel triplex it will result in 5 interruptions (10 overlapping pairs of NIBTs) causing loss of Hoogsteen pairs in 5 out of 14 triplets. Similarly the 36-mer TFO, 5’G**GTGGTG**G***GTTG***GG**GTGGTGGTGTGGTGGTGGTGT**T3' (10 interruptions with 19 overlapping pairs of NIBTs), and the 38-mer TFO, 5' GGGGGG**GTG**GGG**GTGT**TTGG**GTGGTGTGGTG**GGG**GTG**G 3' (8 interruptions with 15 overlapping pairs of NIBTs) targeted against the promoter region of human epidermal growth factor receptor gene and the transcription start site of mouse insulin receptor gene respectively, form only antiparallel triplexes [78] to effect transcription inhibition. Their inability to form parallel triplex is obvious in view of expected loss of Hoogsteen pairs at all the interruption sites (9 out of 36 in 36-mer TFO and 8 out of 38 in 38-mer TFO) making the triplex less viable due to lower stability.

On the other hand, non-overlapping NIBTs in 5’TTTTCmTTTTGGGGGG (one TG step junction) enables it to form a stable triplex with the oligopurine sequence flanking the SV40 origin of replication containing plasmid in COS-1 cells to stall replication [76]. Likewise, a stable parallel triplex [81] is formed with the TFO 5’ GGGG*GT*TTTCTTTT 3', in spite of GT step triplex junction (a pair of non-overlapping NIBTs) which facilitates retention of Hoogsteen pair. This finding that triplex junctions (non-overlapping NIBTs) do not significantly influence triplex formation or its stability gains further support from the observation that the 5' TTTTTGGTTTTTGG 3' forms a triplex in both parallel and antiparallel orientation with a T_m_ of ~ 46°C [80]. Likewise, parallel and antiparallel triplex formed by 5' GGGGTTTTGGGG 3' with 2 triplex junctions (2 sets of non-overlapping pairs of NIBTs) display a similar T_m_ of 57°C & 58°C [72]. But, T_m_ is lowered by ~ 10°C for the parallel triplex formed by the TFO 5’ GGTT**TGT**TT**TGT**TT 3’ compared to its antiparallel counterpart triplex formed by the TFO 5’ TT**TGT**TT**TGT**TTGG 3’. This can be readily attributed to the loss of 2 (out of 14) canonical Hoogsteen hydrogen bonds at the two G_*_GC interruptions (4 overlapping NIBTs) in the parallel triplex [80]. In the antiparallel triplex, reverse Hoogsteen hydrogen bonds are preserved since minor effects of low residual twist of Δt = 10.6° are absorbed by the inherent triplex flexibility. Thus, there exists a direct bearing between the experimental observations and the number and type of NIBTs with MD simulations clearly bringing out the criticality of the mechanistic attributes of base triplet nonisostericity.

Growing evidence for occurrence of nucleic acid triple helices *in vivo* and their implication in diverse critical biological processes, disease and therapy, makes their structural study imperative. Unlike DNA and RNA duplexes where isosteric WC base pairs dominate, nucleic acid triplexes encounter mixture of isosteric and nonisosteric base triplets. Nature and magnitude of base triplet nonisostericity is solely determined by neighbouring triplets. This together with the number of nonisosteric base triplets (NIBTs) determines triplex forming ability, parallel or non-parallel, with or without sequence dependent structural variations as well as their stability. Although base triplet nonisostericity between the parallel G_*_GC & T_*_AT triplets is seemingly obvious [74] quantitative estimates of its nature and magnitude and its mechanistic influence are by no means readily comprehendible. The work presented here fills this critical gap by providing quantitative description of base triplet nonisostericity as well as their mechanistic effects in influencing stability and formation of DNA triplexes. It is found that the extreme base triplet nonisostericity (residual twist) prevalent between the parallel G_*_GC & T_*_AT triplets adds an element of instability, owing to the inevitability of significant stereochemical rearrangements, to the extent of even precluding triplex formation when their incidences are recurrent. Thus, the concept of residual twist has provided a means not only to assess the source and degree of nonisostericity but also has enabled in providing a mechanistic basis for the experimental observations in relation to the feasibility or the lack of same to form parallel DNA triplexes. Although these results are derived from analyses of DNA triplexes they should hold valid in assessing the role of nonisostericity in RNA and RNA.DNA hybrid triplexes as well thus enabling a comprehensive understanding of sequence dependent structural variation in nucleic acid triplexes in general. In fact, this concept has found an application in developing an algorithm to identify triplex forming sequences [90]. The results presented here should aid in designing more specific and efficient TFOs as gene targeting agents (antigene therapeutics). Interestingly, the concept of residual twist proved immensely useful in predicting mechanistic effects of base pair nonisostericity due to juxtaposition of Watson-Crick and different non-Watson-Crick pairs in RNA duplexes [91].

## Materials and Methods

### Triplex model generation

Parallel and anti-parallel DNA triplexes (Sequences 1–19 in Table 1), were built conforming to a 12-fold helix [92] with stereochemistry of the third strand regularized by constrained-restrained molecular geometry optimization and van der Waals energy minimization using X-PLOR [93]. Generated models were then subjected to steepest descent energy minimization using the Sander module of AMBER 12.0 [94] and were used as starting model for MD simulation.

### MD simulation

MD simulation is a technique employed to obtain the dynamical aspects of a protein or nucleic acid structure obtained from X-ray, NMR or modelling investigations. AMBER [94] and CHARMM [95] are the widely used force-fields. Modified version of the Cornell et al. force field [96] as in parmbsc0 and its minor variations in the AMBER [97–100] has provided not only a decisive stabilization of nucleic acid simulations, but also have been able to reproduce with reasonable accuracy several structural and dynamical features of BDNA and non BDNA structures which includes detailed insights into BDNA↔ADNA [101], B→ZDNA transition [102,103], base flipping [104], DNA bending [105] and deformation of DNA/RNA in the presence of proteins [106] etc. AMBER has also been successfully utilised to study properties like stability, folding pathway, role of ions, interaction with drugs and hydration property of various non-BDNA structures like RNA [91], DNA.RNA hybrids [97,107], t-RNA [108,109], i-motif [110], Holliday junction [111], PNA.DNA [112], G-quadruplexes [113,114] etc.

Likewise, MD simulations [115] using AMBER force field bring out the feature intermediate to that of A and B DNA of the duplex in a triplex in accordance with geometrical stipulation [116] and NMR data [117,118]. Also, it has been competently used to explore conformational flexibility [119], stability [120], hydration [121], protonation and folding property [122] etc. of DNA triplexes. In fact, our own earlier investigations on antiparallel triplexes bought out base triplet nonisosteric effects [82, 83]. Given its established utility, AMBER force field is used in the present study of parallel triple helices to enable comparison with antiparallel triplexes.

All MD simulations were performed using AMBER 12.0 program [94]. Triplexes were solvated in a periodic box of TIP3P waters and net-neutralized with Na+ counter ions using Leap module of AMBER Tools 13.0 [123]. Following this, initial equilibration minimization was carried out using steepest descent (2000 cycles) and conjugate-gradient (500 cycles) algorithms, with a positional restraint of 500 kcal/mol Å^2^ on the solute atoms. Further minimization was carried out with positional restraints on the solute reduced in steps of 100 kcal/mol Å^2^. Subsequently, minimization was effected for 1000 cycles without any positional restraints. MD simulation was then carried out in an NVT ensemble, during which the entire system was heated from 0 to 300K over 100 ps. Further equilibration MD was carried out for 200 ps under constant pressure (NPT). Following this, production run MD simulations were initiated for 250 ns for Sequence 1 and 100 ns each for triplex sequences in Sequences 2–19 (Table 1). Equilibration and production run simulations were carried out using the Sander and PMEMD module (optimized for CUDA) of AMBER 12.0 (ff99+parmbsc0) respectively. Periodic boundary conditions and PME under isothermal isobaric conditions (T = 298 K; P = 1 atm) with an integration time step of 2 fs was employed in the simulation. All bonds involving hydrogen were constrained using SHAKE algorithm (tolerance = 0.0005 Å) [124]. Trajectories were analyzed using Ptraj module of Amber Tools 13.0. Base pair, base step parameters and backbone torsion angles were extracted from the output of 3DNA Ver 2.1 [125] using in-house programs. Helical twist angles were calculated with respect to C1’…C1’ vector due to the presence of nonisosteric base pairs [82, 83]. Hydrogen bond distances and angles were computed using the criteria of donor…acceptor distance of < 3.6 Å and hydrogen bond angle >120°. Figures were prepared either using PyMOL (ww.pymol.org) or graphical interface of insight II [126].

### Description of residual twist (Δt°) and radial difference (Δr Å)

Residual twist (Δt°) is calculated by measuring the angle between the line joining C1′ …C1′ atoms of the Hoogsteen/Reverse Hoogsteen pair of the superimposed base triples prior to the application of helical twist angle (t) requisite to generate a triple helical structure. Radial difference (Δr Å) corresponds to half the difference between diameters of the G_*_GC (14.5 Å) & T_*_AT (15.3 Å) base triplets.

### Binding free energy calculations

Binding free energy (ΔG _Bind_) of the triplex structures is estimated by using MM-PBSA [127, 128] module of AMBER 12.0. It is calculated as the binding free energy difference between the bound (triplex) and the unbound (WC duplex and TFO) states.

$${\Delta \text{G}}_{\text{(bind)}}{\;=\text{ G}}_{\left(\text{Triplex}\right)}\;-{\;(\text{G}}_{\left(\text{WC duplex}\right)}{\;+\text{ G}}_{\left(\text{TFO}\right)}\text{)}$$

G _Triplex_, G _WC duplex_ & G_TFO_ are individually calculated by post-processing of the simulation trajectories taken at 20 ps interval after removing counter ions and water molecules. Free energy (G) for each component is given by; G = ΔE_(gas)_−ΔG_(sol)_−TΔS_(gas)_. The gas-phase energy ΔE^gas^ is calculated as summation over the bond length, bond angle, dihedral angle, van der Waals and electrostatics energy contributions. ΔG^solv^ is calculated by summing the polar (G_PB_) and non-polar (G_nonpol_, _sol_) contributions. G_PB_ is calculated by the Poisson-Boltzmann (PB) equation with a dielectric constant value of 1.0 and 80.0 set for solute and solvent respectively. Non-polar contribution G_nonpol’sol_ = γ*SASA, wherein SASA stands for solvent accessible surface area. Value of γ corresponds to 0.0072 kcal/mol. Using normal mode analysis, entropy term (TΔS) is calculated as the sum of translational, rotational and vibrational components. For the normal mode calculation distance-dependent dielectric constant energy minimization is used with ε = 4r and convergence value of 0.5. Terminal triplet at the 5’ and 3’ ends of the triplexes is not considered due to end-effects.

## Supporting Information

S1 Fig Pronounced intrinsic nonisostericity between G_*_GC and T_*_AT triplets leads to nonuniform triplex. (**A**) Illustration of how the residual twist between the nonisosteric G_*_GC (green) and T_*_AT (brown) triplets lead to skewed displacement of the C1’ atoms of the Hoogsteen bases implying the need for large conformational changes in triplex. Note that the C1’ atoms of WC duplex are aligned vertically. Base triplets are related to each other by t = 0° and h = 3.26 Å. (**B**) Stereo view of a G_*_GC triplet sandwiched between T_*_AT triplets (S1A Fig) demonstrates that the skewed arrangement of C1’ atoms disconnects it to the extent of nearly one nucleotide length at both the GT & TG steps, besides causing steric overlap at TG step due to close proximity of adjacent sugar residues. WC duplex and TFO are coloured blue and red respectively. Hydrogen bond is denoted by dashed line (green).
(TIF)

S2 Fig Disruption of canonical Hoogsteen hydrogen bond in G interrupts in a T_*_AT triplex. Average canonical Hoogsteen hydrogen bond distance corresponding to T…A pairs in a (**A**) poly T_*_AT triplex (Sequence 3), (**B**) with a single G interruption (Sequence 4), (**C**) with 2 G interruptions (Sequence 5) and (**D**) with 3 G interruptions (Sequence 6). Standard deviation w.r.to mean distance is indicated above the bar. G interruptions are marked by dashed blue rectangle. Note the large fluctuation in flanking T…A triplets with increase in G interruption in C & D (denoted by orange circle).
(TIF)

S3 Fig Variation in groove widths. Changes in minor grove (m), CH groove (CH) and WH groove (WH) widths in different triplexes: T_*_AT triplex (Sequence 4) with a G_*_GC interruption **(A**); G_*_GC triplex (Sequence 8 with a T_*_AT interruption (**B**); a triplex (Sequence 10) with a GT step junction interface **(C)**; a triplex (Sequence 11) with a TG step junction interface **(D)**. Groove widths corresponding to starting model (thick black line) and average structure (dashed line) calculated for the last 5 ns are shown.
(TIF)

S4 Fig Canonical Hoogsteen hydrogen bond variation in G_*_GC triplex with T interrupts. Average canonical Hoogsteen hydrogen bond distance of G…G pairs constituting a (**A**) poly G_*_GC triplex (Sequence 7), (**B**) with a single T…A interruption (Sequence 8) and (**C**) with 2 T…A interruptions (Sequence 9). Standard deviation w.r.to mean distance is indicated above the bar. Intervening T…A pair are marked by dashed blue rectangle. Note the less fluctuation in flanking T…A triplets with increase in G interruption in C & D (denoted by orange circle) as compared to S3 Fig.
(TIF)

S5 Fig Stable nature of antiparallel G_*_GC triplex with T_*_AT interruption. Frequency of incidence (red & gray colour filled part) and loss (void part) of reverse Hoogsteen hydrogen bonds in the T_*_AT interruption of the G_*_GC triplex (Sequence 17). Conservation (filled part) of canonical hydrogen bonds O2…N6 in and N1…N7 the interrupting T_*_AT triplet is conspicuous (blue box).
(TIF)

S6 Fig Progressive destablisation of triplex with increasing number of overlapping NIBTs. Variation of binding free energy w.r.t triplexes comprising different number of G_*_GC (green) and T_*_AT (red) interruptions (overlapping pair of NIBTs). Sequences 3 & 7 (zero interruption); Sequences 4 & 8 (1 interruption); Sequences 5 & 9 (2 interruptions); Sequence 6 (3 interruptions). Proportional decrease in energy with increase in overlapping NIBTs denotes a linear correlation (indicated by least square line fit).
(TIF)

S7 Fig Large variation of X-displacement at the non-overlapping GT step junction. Illustration of large X-displacement (dashed line) of base pairs of WC duplex near GT junction interfaces in different triplexes viz., (**A**) Sequence 10; (**B**) Sequence 12; (**C**) Sequence 13; (**D**) Sequence 14. X-displacement corresponding to the starting model is depicted as thick black line.
(TIF)

S8 Fig Helical twist and stacking in and around the GT & TG junction. Twist angle variation at the WC T_7_C_8_ (black) and WH G_37_T_38_ (red) steps in the junction triplex with GT interface—Sequence 10 (**A**); at the WC C_8_T_9_ (black) T_7_C_8_ and WH T_37_G_38_ (red) steps in the junction triplex with TG interface- Sequence 11 (**B**). Nature of base stacking in and around the neighbourhood of junction interface in Sequence 10 (**C**); and in Sequence 11 (**D**). Minimal stacking is indicated by arrows. C1' atom of the sugar is shown as open circle.
(TIF)

S9 Fig BI to BII transition at the non-overlapping TG step junction. Variation of backbone torsion angles around the C3’- O3’ (ε; black) and P-O3’ bonds (ζ; red) in different triplexes (Sequences 10–14). Note the switch from BI to BII conformation at the TG step in Sequences 11–14 (indicated by arrow). The GT step assumes the preferred BI conformation.
(TIF)

S10 Fig Kinked triplexes. Snapshot of parallel triplexes showing a curvature near the neighbourhood of GT step: Sequence 12 (**A**); Sequence 13 (**B**): and Sequence 14 (**C**). G_*_GC and T_*_AT mini triplexes are coloured green and red respectively. Bent curvature is indicated by a black circle. 5’- terminus of TFO is indicated. Helical axis of WC duplex of the triplex is shown (blue sphere).
(TIF)

## Abbreviations

G_W_, A_W_, T_W_ and C_W_: Guanine, cytosine, adenine and thymine bases of the WC duplex
G_H_ and T_H_: Guanine and thymine bases of the parallel Hoogsteen strand (third strand)
G_RH_ and T_RH_: Guanine and thymine bases of the antiparallel reverse Hoogsteen strand (third strand)
TFOs: Triplex forming oligonucleotides
