# Correction: Selective Preference of Parallel DNA Triplexes Is Due to the Disruption of Hoogsteen Hydrogen Bonds Caused by the Severe Nonisostericity between the G*GC and T*AT Triplets

**DOI:** 10.1371/journal.pone.0155090

**Published:** 2016-05-06

**Authors:** Gunaseelan Goldsmith, Thenmalarchelvi Rathinavelan, Narayanarao Yathindra

There are errors within Figs 2, 4 and 6. The titles of the graphs are incorrect. The word ‘Scheme’ should be ‘Sequence’.

**Fig 2:**
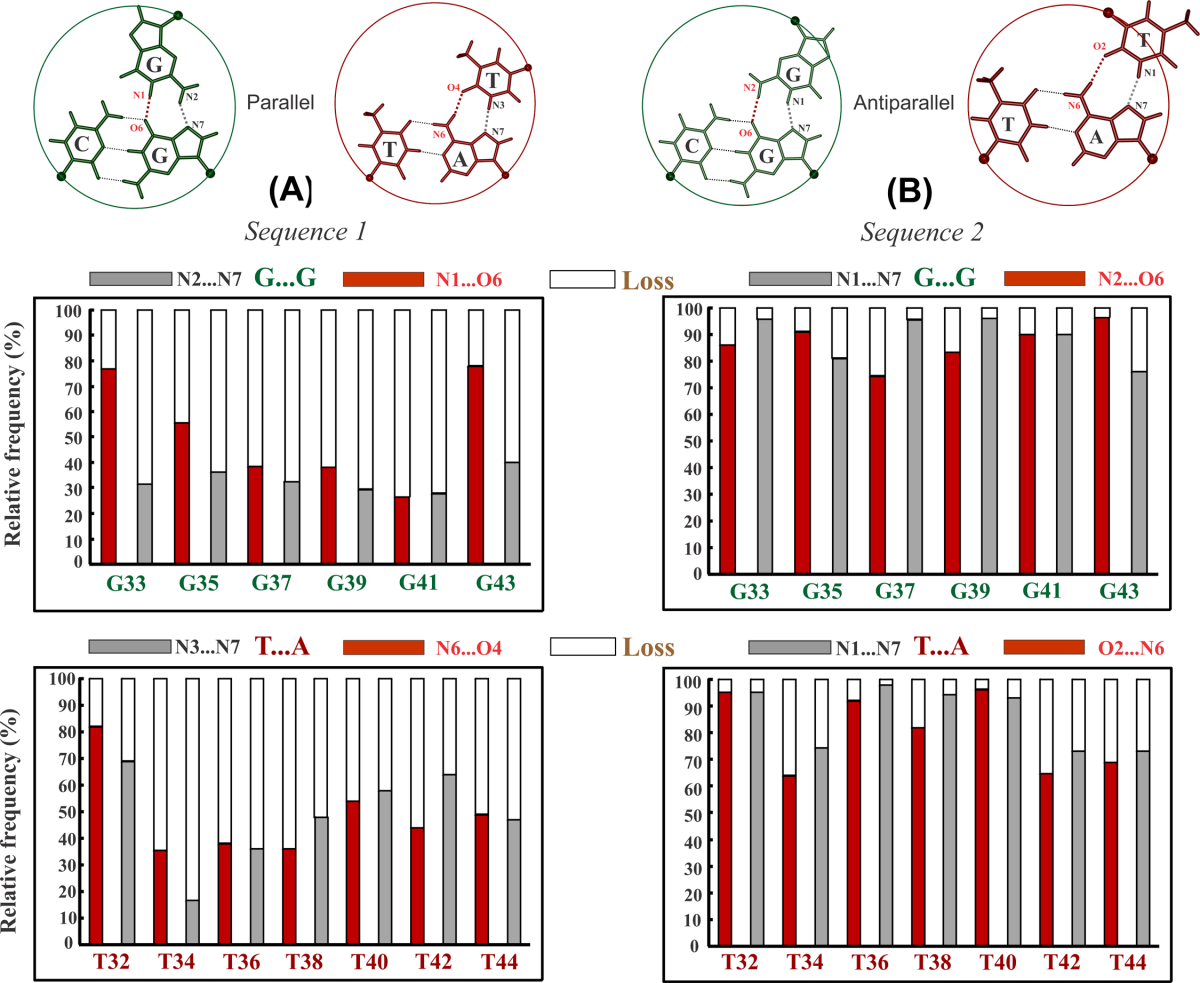
Demonstration of unstable and stable nature of parallel and antiparallel triplex formed by alternating G*GC & T*AT triplets respectively. A Frequency of incidence (red & gray filled part) and loss (void part) of canonical (A) Hoogsteen (Sequence 1) and (B) reverse Hoogsteen hydrogen bonds (Sequence 2) in the central 6 G*GC and 7 T*AT triplets of the 15-mer parallel and antiparallel triplex (terminal triplets not considered) over a simulation time of 250 and 100 ns respectively. Wide spread loss of Hoogsteen hydrogen bonds in the (A) parallel triplex (Sequence 1), and retention of reverse Hoogsteen hydrogen bonds in the (B) antiparallel triplex (Sequence 2) are apparent. Canonical Hoogsteen and reverse Hoogsteen hydrogen bonding schemes in the G*GC & T*AT base triplets in parallel & antiparallel orientations are shown on top for reference.

**Fig 4:**
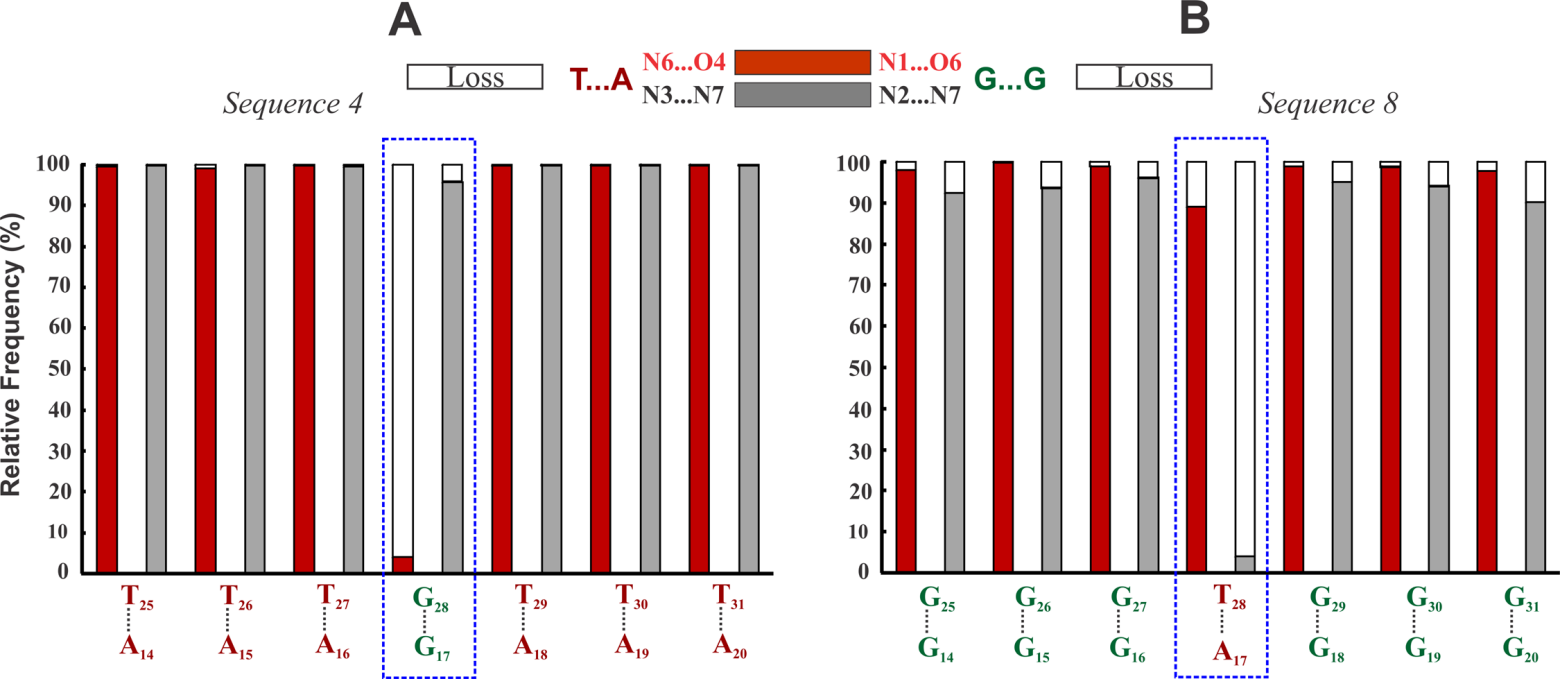
Destabilization of canonical Hoogsteen hydrogen bond in the G*GC and T*AT interruptions. Frequency of incidence (red & gray filled part) and loss (void part) of Hoogsteen hydrogen bonds in the G*GC interruption of the T*AT triplex (A) (Sequence 4) and in the T*AT interruption of the G*GC triplex (B) (Sequence 8). Loss of the canonical Hoogsteen hydrogen bond (void part) in the interrupting G*GC (N1…O6) and T*AT (N3…N7) triplets are conspicuous (blue box).

**Fig 6:**
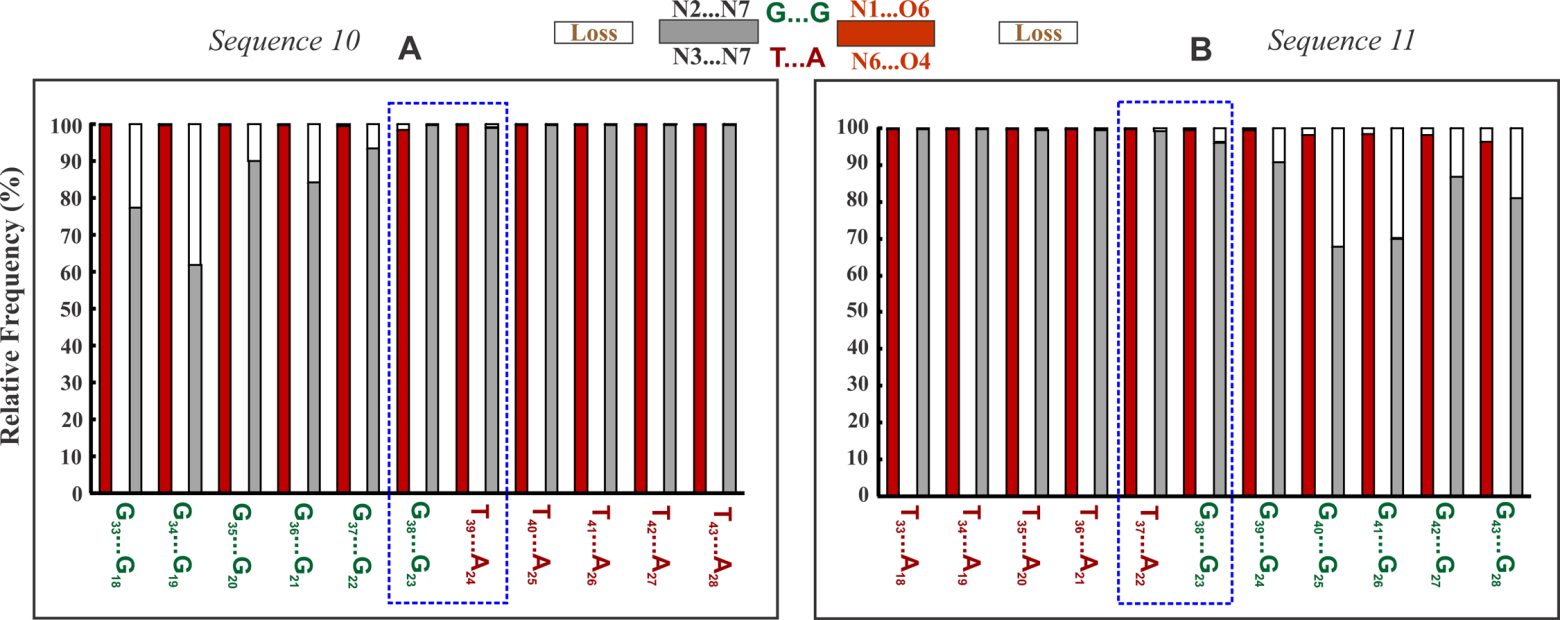
Demonstration of the retention of Canonical Hoogsteen hydrogen bonds at the G*GC/T*AT triplex junction interface. Frequency of incidence (red & gray colour part) and loss (void part) of Hoogsteen hydrogen bonds in the NIBTs at the GT step—Sequence 10 (A) and at the TG step—Sequence 11 (B) triplex junction interfaces. Hydrogen bonds retention is highlighted by the enclosed blue box.

There are errors within Supporting Information files 2, 3, 4, 5, 7, 8, 9 and 10. The word ‘Scheme’ should be ‘Sequence’.

## Supporting Information

S2 Fig Disruption of canonical Hoogsteen hydrogen bond in G interrupts in a T*AT triplex. Average canonical Hoogsteen hydrogen bond distance corresponding to T…A pairs in a (A) poly T*AT triplex (Sequence 3), (B) with a single G interruption (Sequence 4), (C) with 2 G interruptions (Sequence 5) and (D) with 3 G interruptions (Sequence 6). Standard deviation w.r.to mean distance is indicated above the bar. G interruptions are marked by dashed blue rectangle. Note the large fluctuation in flanking T…A triplets with increase in G interruption in C & D (denoted by orange circle).
(TIF)

S3 Fig Variation in groove widths. Changes in minor grove (m), CH groove (CH) and WH groove (WH) widths in different triplexes: T*AT triplex (Sequence 4) with a G*GC interruption (A); G*GC triplex (Sequence 8 with a T*AT interruption (B); a triplex (Sequence 10) with a GT step junction interface (C); a triplex (Sequence 11) with a TG step junction interface (D). Groove widths corresponding to starting model (thick black line) and average structure (dashed line) calculated for the last 5 ns are shown.
(TIF)

S4 Fig Canonical Hoogsteen hydrogen bond variation in G*GC triplex with T interrupts. Average canonical Hoogsteen hydrogen bond distance of G…G pairs constituting a (A) poly G*GC triplex (Sequence 7), (B) with a single T…A interruption (Sequence 8) and (C) with 2 T…A interruptions (Sequence 9). Standard deviation w.r.to mean distance is indicated above the bar. Intervening T…A pair are marked by dashed blue rectangle. Note the less fluctuation in flanking T…A triplets with increase in G interruption in C & D (denoted by orange circle) as compared to S3 Fig.
(TIF)

S5 Fig Stable nature of antiparallel G*GC triplex with T*AT interruption. Frequency of incidence (red & gray colour filled part) and loss (void part) of reverse Hoogsteen hydrogen bonds in the T*AT interruption of the G*GC triplex (Sequence 17). Conservation (filled part) of canonical hydrogen bonds O2…N6 in and N1…N7 the interrupting T*AT triplet is conspicuous (blue box).
(TIF)

S7 Fig Large variation of X-displacement at the non-overlapping GT step junction. Illustration of large X-displacement (dashed line) of base pairs of WC duplex near GT junction interfaces in different triplexes viz., (A) Sequence 10; (B) Sequence 12; (C) Sequence 13; (D) Sequence 14. X-displacement corresponding to the starting model is depicted as thick black line.
(TIF)

S8 Fig Helical twist and stacking in and around the GT & TG junction. Twist angle variation at the WC T7C8 (black) and WH G37T38 (red) steps in the junction triplex with GT interface—Sequence 10 (A); at the WC C8T9 (black) T7C8 and WH T37G38 (red) steps in the junction triplex with TG interface- Sequence 11 (B). Nature of base stacking in and around the neighbourhood of junction interface in Sequence 10 (C); and in Sequence 11 (D). Minimal stacking is indicated by arrows. C1' atom of the sugar is shown as open circle.
(TIF)

S9 Fig BI to BII transition at the non-overlapping TG step junction. Variation of backbone torsion angles around the C3’- O3’ (ε; black) and P-O3’ bonds (ζ; red) in different triplexes (Sequences 10–14). Note the switch from BI to BII conformation at the TG step in Sequences 11–14 (indicated by arrow). The GT step assumes the preferred BI conformation.
(TIF)

S10 Fig Kinked triplexes. Snapshot of parallel triplexes showing a curvature near the neighbourhood of GT step: Sequence 12 (A); Sequence 13 (B): and Sequence 14 (C). G*GC and T*AT mini triplexes are coloured green and red respectively. Bent curvature is indicated by a black circle. 5’- terminus of TFO is indicated. Helical axis of WC duplex of the triplex is shown (blue sphere).
(TIF)
