# Supplementary figures and images for: Correction: Selective Preference of Parallel DNA Triplexes Is Due to the Disruption of Hoogsteen Hydrogen Bonds Caused by the Severe Nonisostericity between the G*GC and T*AT Triplets
Source: PLoS One. 2016 May 6;11(5):e0155090. doi: 10.1371/journal.pone.0155090 (PMC4859481; doi:10.1371/journal.pone.0155090)

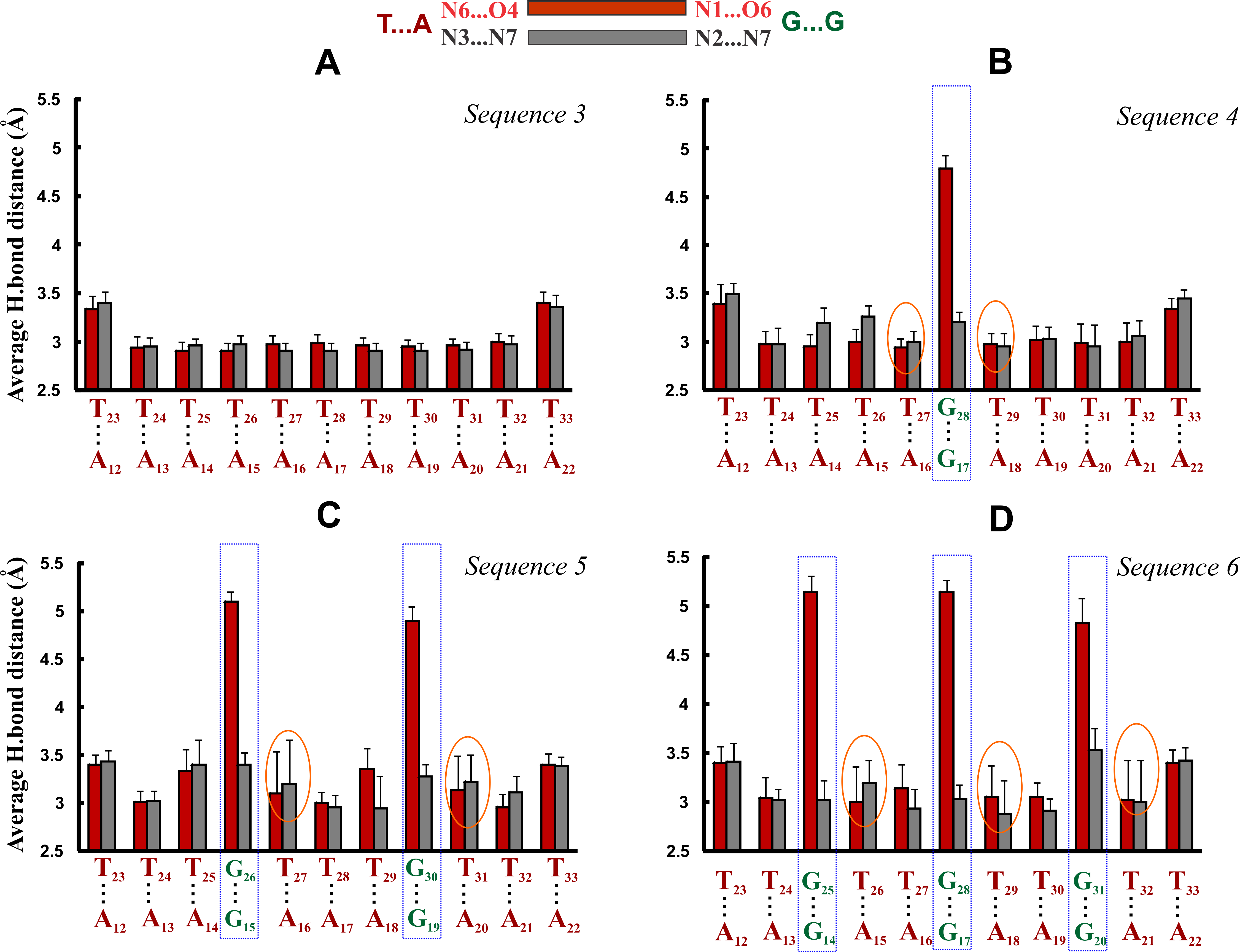

Supplement: S2 Fig — Average canonical Hoogsteen hydrogen bond distance corresponding to T…A pairs in a (A) poly T*AT triplex (Sequence 3), (B) with a single G interruption (Sequence 4), (C) with 2 G interruptions (Sequence 5) and (D) with 3 G interruptions (Sequence 6). Standard deviation w.r.to mean distance is indicated above the bar. G interruptions are marked by dashed blue rectangle. Note the large fluctuation in flanking T…A triplets with increase in G interruption in C & D (denoted by orange circle). (TIF) [file pone.0155090.s001.tif]

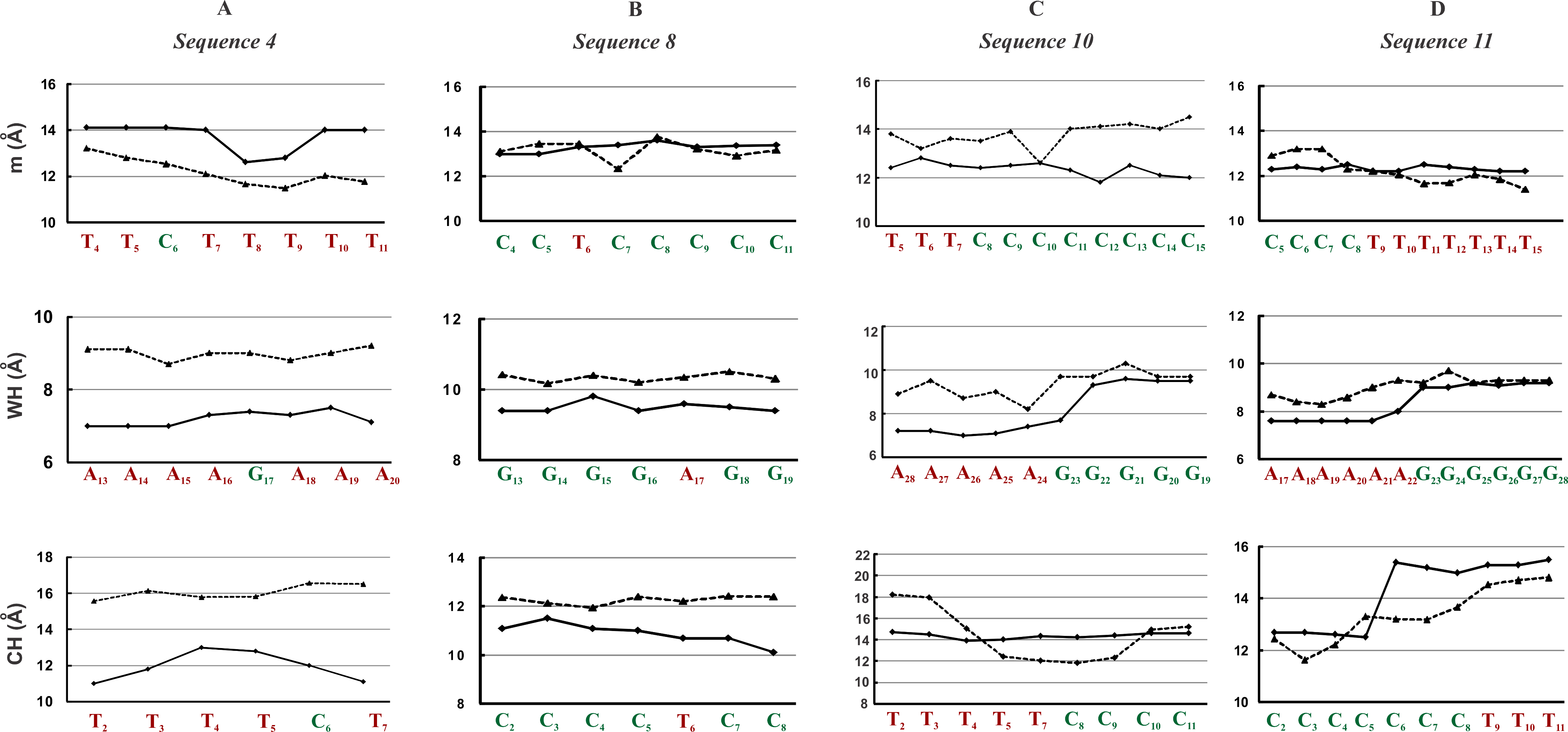

Supplement: S3 Fig — Changes in minor grove (m), CH groove (CH) and WH groove (WH) widths in different triplexes: T*AT triplex (Sequence 4) with a G*GC interruption (A); G*GC triplex (Sequence 8 with a T*AT interruption (B); a triplex (Sequence 10) with a GT step junction interface (C); a triplex (Sequence 11) with a TG step junction interface (D). Groove widths corresponding to starting model (thick black line) and average structure (dashed line) calculated for the last 5 ns are shown. (TIF) [file pone.0155090.s002.tif]

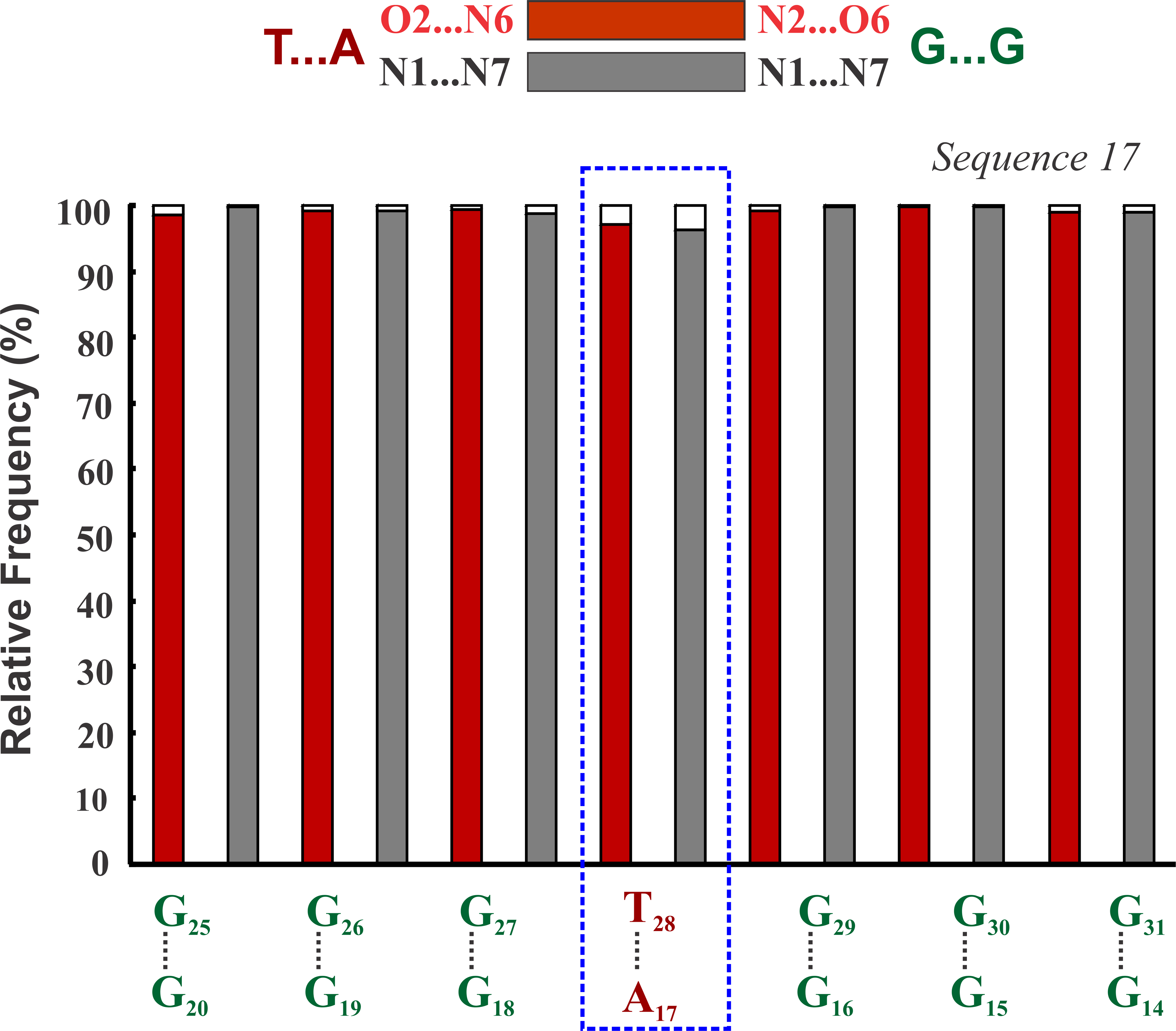

Supplement: S5 Fig — Frequency of incidence (red & gray colour filled part) and loss (void part) of reverse Hoogsteen hydrogen bonds in the T*AT interruption of the G*GC triplex (Sequence 17). Conservation (filled part) of canonical hydrogen bonds O2…N6 in and N1…N7 the interrupting T*AT triplet is conspicuous (blue box). (TIF) [file pone.0155090.s004.tif]

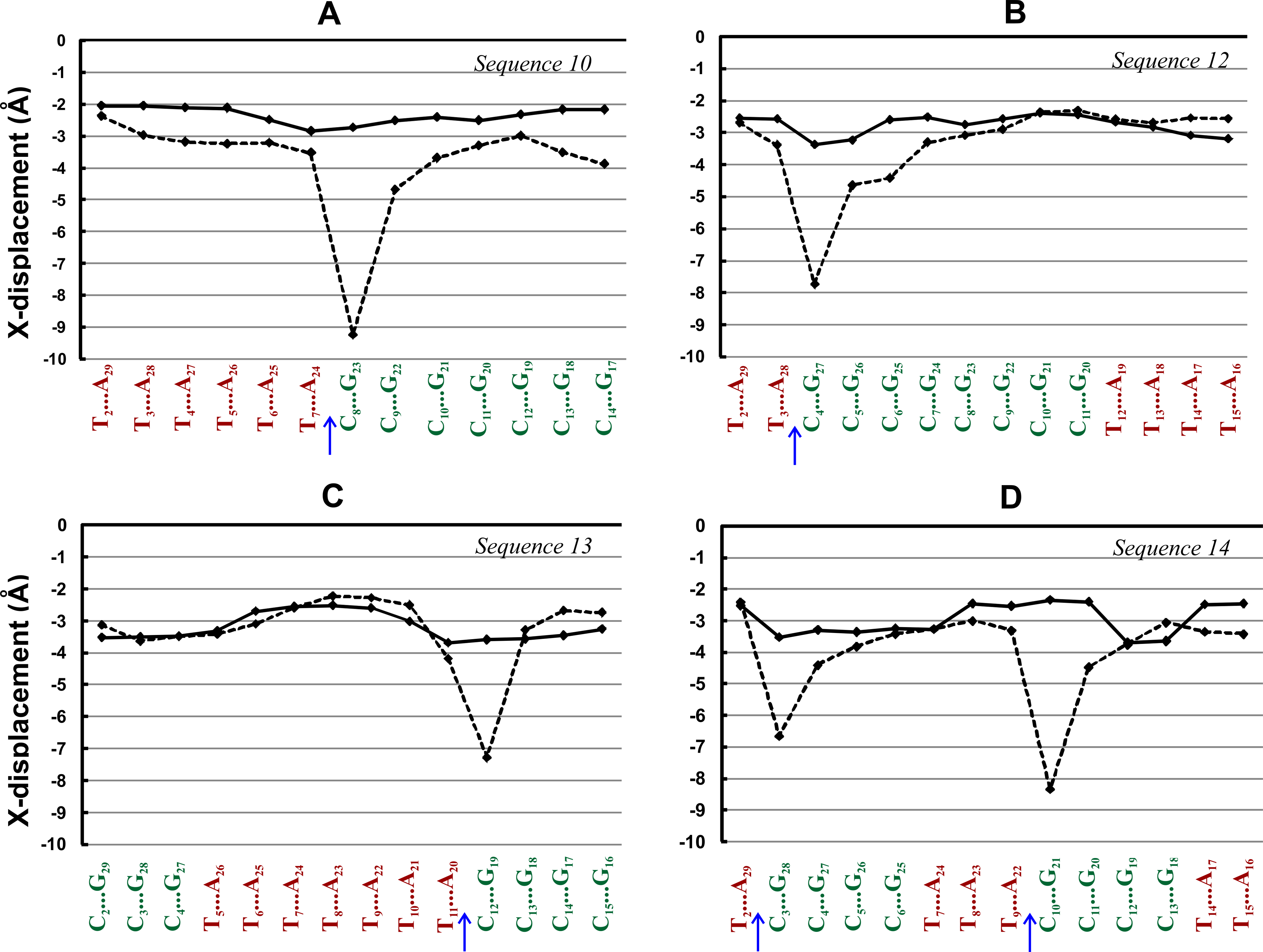

Supplement: S7 Fig — Illustration of large X-displacement (dashed line) of base pairs of WC duplex near GT junction interfaces in different triplexes viz., (A) Sequence 10; (B) Sequence 12; (C) Sequence 13; (D) Sequence 14. X-displacement corresponding to the starting model is depicted as thick black line. (TIF) [file pone.0155090.s005.tif]

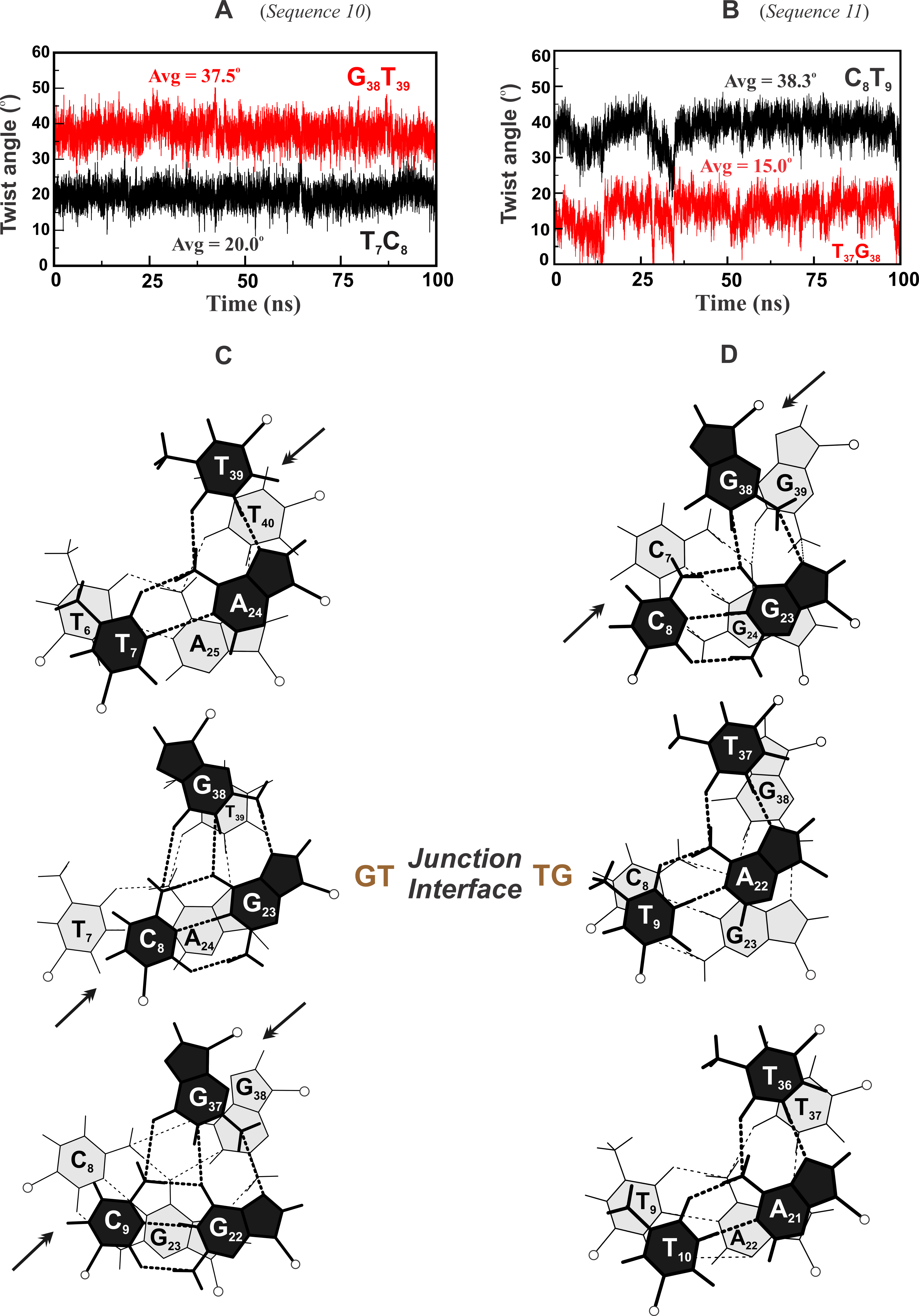

Supplement: S8 Fig — Twist angle variation at the WC T7C8 (black) and WH G37T38 (red) steps in the junction triplex with GT interface—Sequence 10 (A); at the WC C8T9 (black) T7C8 and WH T37G38 (red) steps in the junction triplex with TG interface- Sequence 11 (B). Nature of base stacking in and around the neighbourhood of junction interface in Sequence 10 (C); and in Sequence 11 (D). Minimal stacking is indicated by arrows. C1' atom of the sugar is shown as open circle. (TIF) [file pone.0155090.s006.tif]

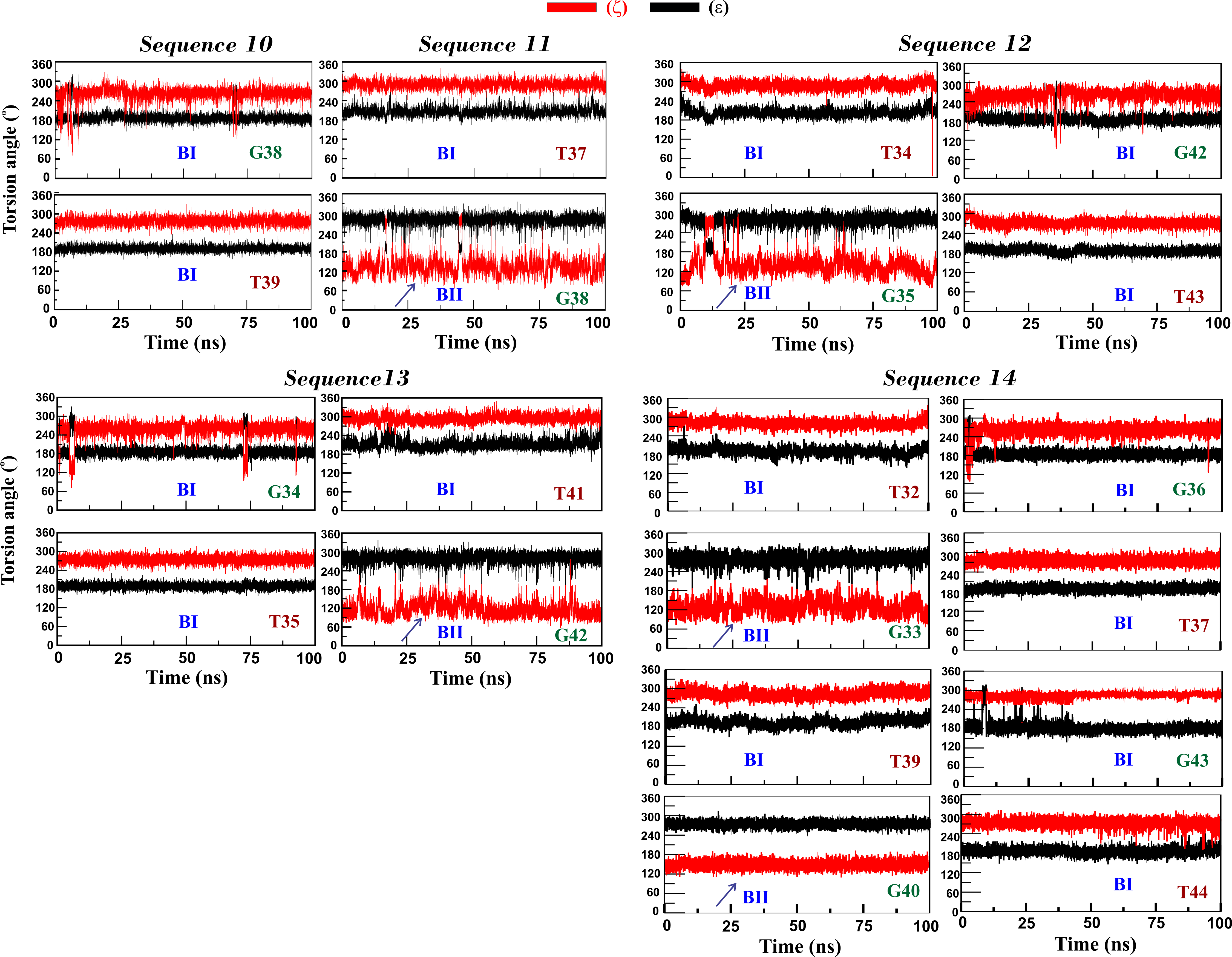

Supplement: S9 Fig — Variation of backbone torsion angles around the C3’- O3’ (ε; black) and P-O3’ bonds (ζ; red) in different triplexes (Sequences 10–14). Note the switch from BI to BII conformation at the TG step in Sequences 11–14 (indicated by arrow). The GT step assumes the preferred BI conformation. (TIF) [file pone.0155090.s007.tif]

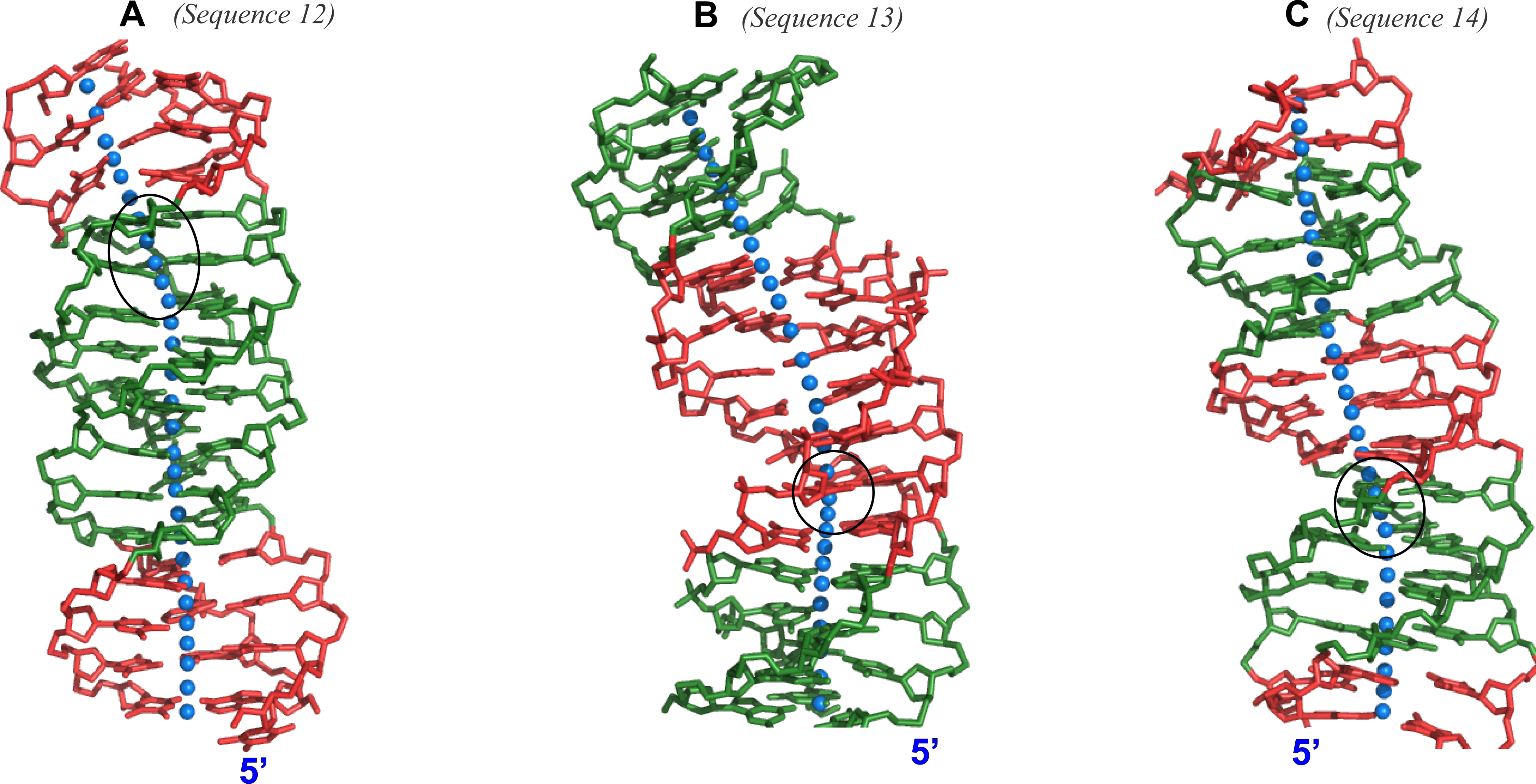

Supplement: S10 Fig — Snapshot of parallel triplexes showing a curvature near the neighbourhood of GT step: Sequence 12 (A); Sequence 13 (B): and Sequence 14 (C). G*GC and T*AT mini triplexes are coloured green and red respectively. Bent curvature is indicated by a black circle. 5’- terminus of TFO is indicated. Helical axis of WC duplex of the triplex is shown (blue sphere). (TIF) [file pone.0155090.s008.tif]
